# Novel live cell fluorescent probe for human-induced pluripotent stem cells highlights early reprogramming population

**DOI:** 10.1186/s13287-021-02171-6

**Published:** 2021-02-05

**Authors:** Sandhya Sriram, Nam-Young Kang, Subha Subramanian, Tannistha Nandi, Samydurai Sudhagar, Qiaorui Xing, Gerine Jin-Ling Tong, Allen Kuan-Liang Chen, Thekkeparambil Chandrabose Srijaya, Patrick Tan, Yuin-Han Loh, Young-Tae Chang, Shigeki Sugii

**Affiliations:** 1 Fat Metabolism and Stem Cell Group, Singapore Bioimaging Consortium, A*STAR, 11 Biopolis Way, Singapore, 138667 Singapore; 2 Laboratory of Bioimaging Probe Development, Singapore Bioimaging Consortium, A*STAR, 11 Biopolis Way, Singapore, 138667 Singapore; 3 Department of Creative IT Engineering, Pohang University of Science and Technology (POSTECH), Pohang, 37673 Republic of Korea; 4 Cancer Therapeutics and Stratified Oncology, Genome Institute of Singapore, 60 Biopolis Street, Genome #02-01, Singapore, 138672 Singapore; 5 Genome Institute of Singapore, 60 Biopolis Street, Genome, #02-01, Singapore, 138672 Singapore; 6 Epigenetics and Cell Fates Laboratory, Institute of Molecular and Cell Biology, 61 Biopolis Drive, Singapore, 138673 Singapore; 7 School of Biological Sciences, Nanyang Technological University, Singapore, 637551 Singapore; 8 Bioprocessing Technology Institute, A*STAR, 20 Biopolis Way, #06-01 Centros, Singapore, 138668 Singapore; 9 Department of Restorative Dentistry, Faculty of Dentistry, University of Malaya, 50603 Kuala Lumpur, Malaysia; 10 Duke-NUS Medical School, 8 College Road, Singapore, 169857 Singapore; 11 SingHealth/Duke-NUS Institute of Precision Medicine, Singapore, 168752 Singapore; 12 Department of Biological Sciences, National University of Singapore, 14 Science Drive 4, Singapore, 117543 Singapore; 13 Department of Chemistry, National University of Singapore, 3 Science Drive 3, Singapore, 117543 Singapore; 14 Department of Chemistry, POSTECH, Pohang, Gyeongbuk 37673 Republic of Korea; 15 Center for Self-assembly and Complexity, Institute for Basic Science (IBS), Pohang, 37673 Republic of Korea; 16 Cardiovascular and Metabolic Disorders Program, Duke-NUS Medical School, 8 College Road, Singapore, 169857 Singapore; 17 Institute of Bioengineering and Nanotechnology, A*STAR, 31 Biopolis Way, Singapore, 138669 Singapore

**Keywords:** DOFLA library fluorescence dye, Human induced pluripotent stem cell (hiPSC), Early stage pluripotency, Mesenchymal-epithelial transition (MET), Adipose-derived stromal cell (ASC), Dental pulp stem cell (DPSC), Golgi marker, Three-dimensional (3D) microcarrier-based culture system, Tra-1-60, cAMP responsive element binding protein (CREB)

## Abstract

**Background:**

Despite recent rapid progress in method development and biological understanding of induced pluripotent stem (iPS) cells, there has been a relative shortage of tools that monitor the early reprogramming process into human iPS cells.

**Methods:**

We screened the in-house built fluorescent library compounds that specifically bind human iPS cells. After tertiary screening, the selected probe was analyzed for its ability to detect reprogramming cells in the time-dependent manner using high-content imaging analysis. The probe was compared with conventional dyes in different reprogramming methods, cell types, and cell culture conditions. Cell sorting was performed with the fluorescent probe to analyze the early reprogramming cells for their pluripotent characteristics and genome-wide gene expression signatures by RNA-seq. Finally, the candidate reprogramming factor identified was investigated for its ability to modulate reprogramming efficiency.

**Results:**

We identified a novel BODIPY-derived fluorescent probe, BDL-E5, which detects live human iPS cells at the early reprogramming stage. BDL-E5 can recognize authentic reprogramming cells around 7 days before iPS colonies are formed and stained positive with conventional pluripotent markers. Cell sorting of reprogrammed cells with BDL-E5 allowed generation of an increased number and higher quality of iPS cells. RNA sequencing analysis of BDL-E5-positive versus negative cells revealed early reprogramming patterns of gene expression, which notably included CREB1. Reprogramming efficiency was significantly increased by overexpression of CREB1 and decreased by knockdown of CREB1.

**Conclusion:**

Collectively, BDL-E5 offers a valuable tool for delineating the early reprogramming pathway and clinically applicable commercial production of human iPS cells.

**Supplementary Information:**

The online version contains supplementary material available at 10.1186/s13287-021-02171-6.

## Background

The discovery of human embryonic stem (ES) cell-like-induced pluripotent stem (iPS) cells has revolutionized and accelerated the new development of personalized drug screening, human disease modeling, and regenerative therapeutics [1, 2]. Despite rapid development of methods to derive human iPS cells, there have been several problems and challenges with reprogramming protocols. These include relatively low efficiency of obtaining high-quality cells, long duration of complete reprogramming processes (typically 3–4 weeks before colony formation), and difficulty in prompt analysis and identification of high quality iPS cells [2, 3]. The low efficiency and long-time course worsen when clinically applicable protocols are attempted by adapting non-viral transduction and feeder-free reprogramming methods. By using selective cell types, efficiency and time can be improved. For example, we previously found that adipose-derived stem cells (ASCs) and dental pulp-derived stem cells (DPSCs) allow feeder-free reprogramming with relatively high efficiencies and shorter time frames [4–6]. However, the technology to promptly distinguish authentic pluripotent stem cells from other somatic cell populations is still underdeveloped. Pluripotent gene reporters have been developed [7], but making transgenic cell lines by using reporters is tedious and not widely applicable for diverse ranges of cell types. Using fluorescent dye-conjugated antibodies for pluripotent cell surface markers such as TRA-1-60/81 and SSEA3/4, or fluorescent substrates for alkaline phosphatase is the most common method to detect iPS cells [8]. Nevertheless, alkaline phosphatase and SSEA3/4 are not very specific to bona fide pluripotent stem cells [9] and reported to be detectable in adult stem cells including ASCs and DPSCs [10, 11]. All of these markers typically stain well-developed colonies of iPS cells only, which can be visible and recognized by experienced observers even under phase contrast microscopy. In addition, it is relatively expensive to manufacture these fluorescent probes, which may increase the cost and hinder the clinical and commercial development of iPS technology. We previously identified a small molecule fluorescent compound, named CDy1, that was screened against mouse ES and iPS cells [12, 13]. CDy1 allowed early stage live cell staining and sorting of reprogramming cells. Here, we report the identification of a novel fluorescent probe, BDL-E5, which specifically detects human iPS cells at the early reprogramming stage. BDL-E5 detected pluripotent cells around 7 days before iPS colonies were visible and stained with Tra-1-60. BDL-E5-sorted reprogramming cells exhibited efficient iPS colony formation. Comprehensive gene expression analysis by RNA sequencing indicated characteristic changes to genes, including *CREB1* and other factors. We subsequently found that cAMP responsive element binding protein (CREB1) plays a role in the reprogramming process into human iPS cells.

## Results

### Screening for a human pluripotency-specific fluorescent probe

**Fig. 1:**
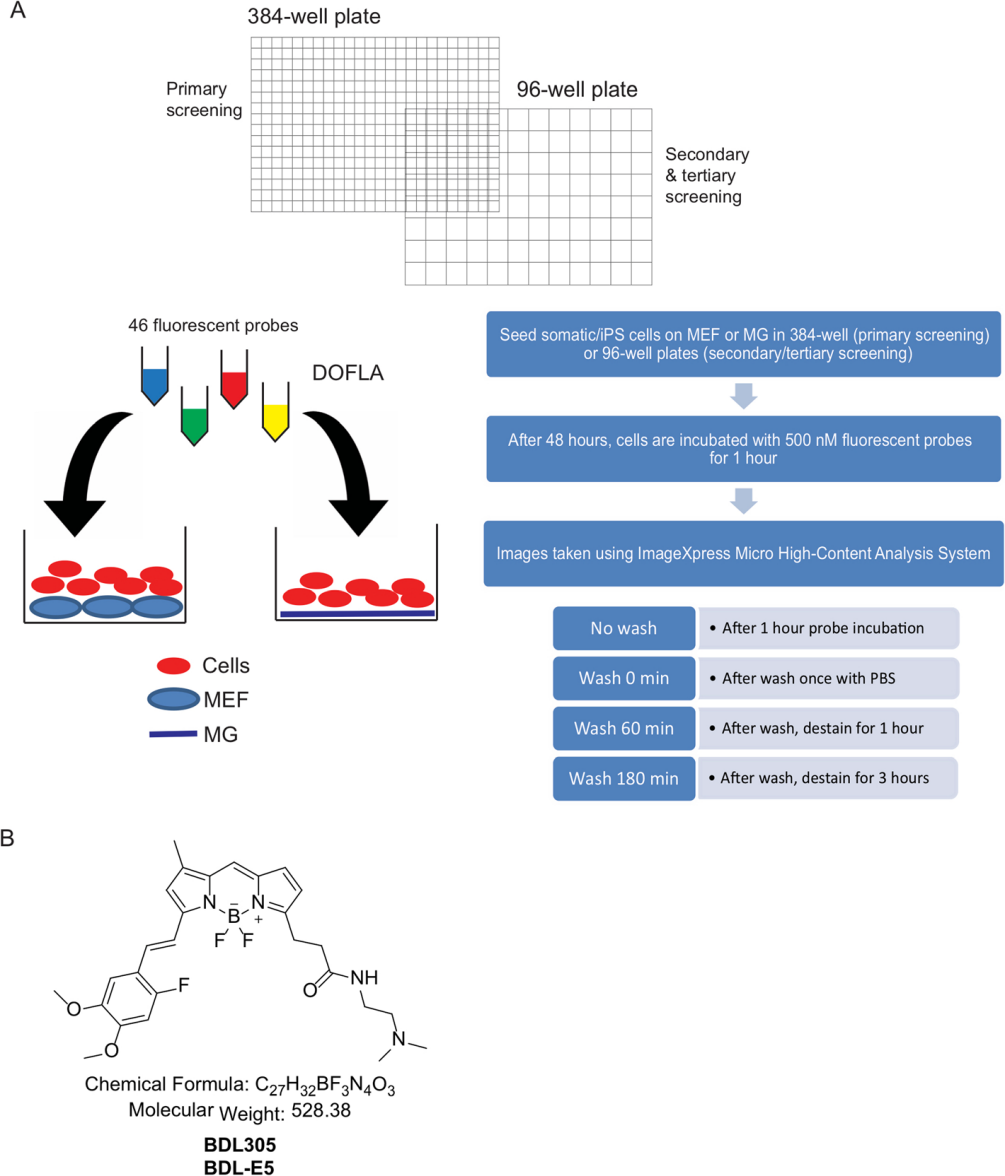
**a** Schematic diagram showing the screening process of fluorescent probes for somatic and iPS cells using DOFLA. Cells were seeded on either MEFs or MG in 384-well plates for primary screening and 96-well plates for secondary and tertiary screening. After 48 h, cells were stained with probes for 1 h and images were taken in the ImageXpress under various conditions as indicated. **b** The chemical structure of the probe, BDL-E5, used in this study

We have previously built > 10,000 fluorescent library compounds for various cell selective imaging probe development, which was named Diversity Orientated Fluorescence Library Approach (DOFLA) [14]. A high-throughput system using the in-house DOFLA [15–17] was employed to screen 46 fluorescent probes that we predicted may specifically recognize pluripotent stem cells. Twenty-nine out of the 46 probes were previously used for screening of mouse ES cells [12, 13]. In this study, we expanded the library, which contains novel compounds mainly harboring rosamine and BODIPY derivatives. To identify fluorescent probes that detected human pluripotent stem cells, ASCs, ASC-derived iPS (AiPS) cells, DPSCs, and DPSC-derived iPS (DiPS) cells were used. ASCs and DPSCs were chosen as these cells show relatively high reprogramming efficiencies and were previously shown to exhibit many of the conventional pluripotent markers, thus serving as stringent negative controls for authentic pluripotent stem cells. Cells were seeded onto 384-well plates (primary screen) or 96-well plates (secondary and tertiary screen) coated with mouse embryonic fibroblasts (MEF) or matrigel (MG) for DOFLA screening (Fig. 1a). After 48 h, cells were stained with library probes (500 nM) for 1 h. Fluorescence was imaged using the ImageXpress System under the following conditions: “no wash” (after 1 h probe incubation), “wash 0 min” (after one wash with PBS), “wash 60 min” (after wash and destain for 1 h), and “wash 180 min” (after wash and destain for 3 h) (Fig. 1a).

**Fig. 2:**
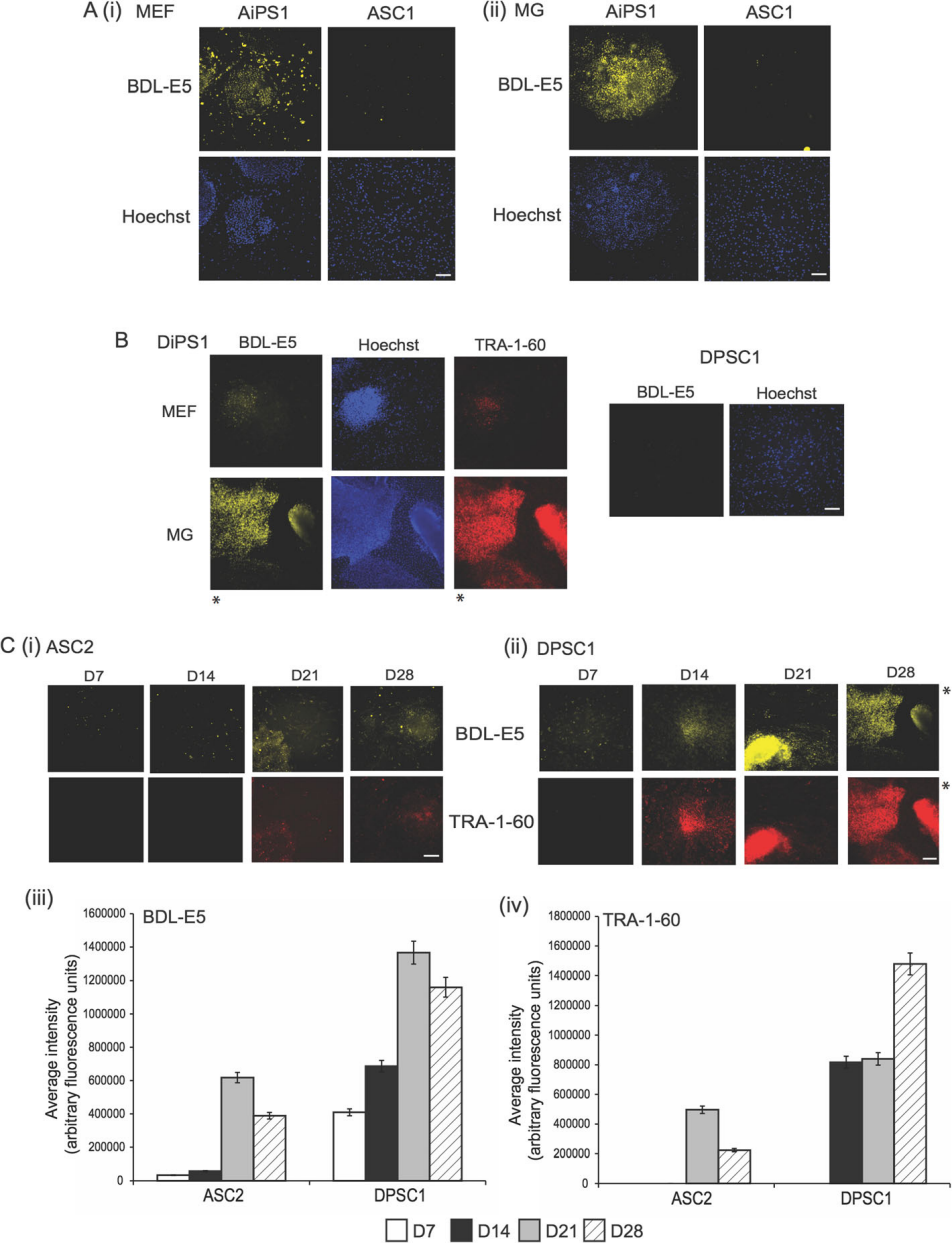
**a** Fluorescent images (10X objective) of BDL-E5 probe (no wash) and Hoechst 33342 staining of AiPS1 colonies and its original ASC1 (ASC line #1) on MEF- (i) and MG-coated (ii) plates from primary screening (*n* = 3). **b** Fluorescent images (10X) of BDL-E5 probe (no wash), Hoechst, and TRA-1-60 on DiPS1 colonies and DPSC1 (DPSC line #1) on MEF- and MG-coated plates from secondary screening (*n* = 3). **c** Fluorescent images (10X) and average fluorescence intensity of BDL-E5 probe (no wash) and TRA-1-60 on ASC2 (i), (iii), and DPSC1 (ii), (iv) on MG-coated plates at 7, 14, 21, and 28 days post nucleofection (dpn) with reprogramming factors. *Represents the same images. Cells were incubated with 500 nM of BDL-E5 in appropriate media for 1 h (*n* = 3). Scale bar represents 100 μm

Images were analyzed using the MetaXpress Image Acquisition and Analysis software. Following tertiary screening, two probes were shortlisted to develop further as pluripotent probes: BDL-E5 and CDy1. CDy1 is among 29 probes that were previously screened against mouse ES cells and was extensively studied [12, 13]. The chemical structure of BDL-E5 is depicted in Fig. 1b. BDL-E5 is based on 4,4-difluoro-4-bora-3a,4a-diaza-s-indacene (BODIPY), with calculated mass of 528.3 and absorption maximum/emission maximum of 578/599 nm. These two probes showed significantly increased intensity of fluorescence in human iPS cells compared with their original somatic cells and MEFs. Figure 2a shows increased BDL-E5 staining (no wash) in AiPS colonies grown on MEF- or MG-coated plates, compared with their original somatic cells from the primary screening. Figure S1 shows increased CDy1 staining in AiPS cells on MEF- or MG-coated plates compared with ASCs from the primary screen.

Secondary screening was performed to confirm that the probes selectively stained different iPS colonies. Figure 2b showed significantly increased fluorescence intensity of BDL-E5 staining (no wash) in DiPS colonies grown on MEF- or MG-coated plates compared with original DPSCs. The BDL-E5^+^ colonies were also positively stained for the pluripotency marker TRA-1-60. Figure S1B showed increased staining of CDy1 and TRA-1-60-positive staining in DiPS colonies grown on MEF- or MG-coated plates compared with DPSCs from the secondary screening. Different classes of probes were shown to highlight human iPS cells in both feeder and feeder-free conditions. These results also confirm applicability of CDy1 to human cells.

### BDL-E5 identified as a live fluorescent probe that detects pluripotent stem cells

Based on the primary and secondary screenings for live fluorescent probes that can specifically identify pluripotent cells, BDL-E5 and CDy1 were chosen as two probes to further analyze. Tertiary screening was performed using the two probes on AiPS colonies under the following probe staining conditions: no wash, wash 0 min, wash 60 min, and wash 180 min. As shown in Fig. S2A, when AiPS colonies were grown on MEF-coated plates, BDL-E5 staining was greatest with regard to the signal-to-background ratio under no wash conditions, and CDy1 staining was greatest under wash 60 min or wash 180 min conditions. When AiPS colonies were grown on MG-coated plates (Fig. S2B), BDL-E5 staining was greatest under no wash conditions, and CDy1 staining was greatest under wash 60 min or wash 180 min conditions. Similar results were also obtained with different subject-derived AiPS colonies, as shown in Fig. S2C.

### BDL-E5 can identify early reprogramming cells

After combining all the results from the primary, secondary, and tertiary screening, the BDL-E5 probe was chosen as the best probe among the screened probes, as it did not require washing (thus was less time- and labor-intensive). Further experiments were carried out using BDL-E5. To determine whether BDL-E5 identifies the early stages of pluripotency, ASCs and DPSCs were reprogrammed using nucleofection of episomal vectors and seeded onto MG-coated plates (feeder-free, viral-free reprogramming method). BDL-E5 staining was performed on reprogramming cells at 7, 14, 21, and 28 days post nucleofection (dpn). As shown in Fig. 2c (i) and (ii), the intensity of the fluorescence of BDL-E5 staining increased with increasing time as iPS colonies were formed from ASCs or DPSCs. TRA-1-60 staining further confirmed that cells that stained positive for BDL-E5 were pluripotent. Quantitative data confirmed this staining as shown in Fig. 2c (iii) and (iv). Interestingly, BDL-E5-positive cells appeared well before colonies were visible and stained positively for TRA-1-60. BDL-E5-positive cells were found around 14 dpn (as opposed to 21 dpn for TRA-1-60-positive colonies) in reprogramming ASCs, while they were observed as early as 7 dpn (as opposed to 14 dpn for TRA-1-60) in reprogramming DPSCs.

**Fig. 3:**
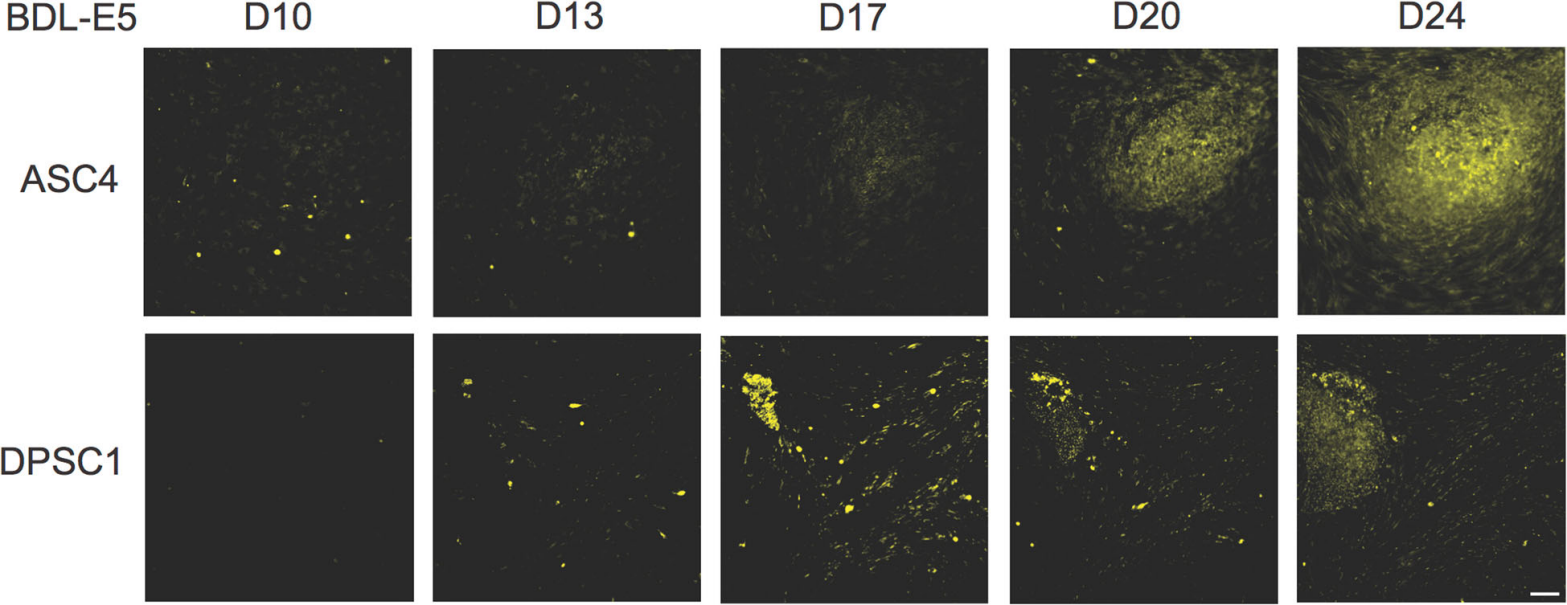
Images showing BDL-E5 staining tracked daily on reprogrammed cells of ASC4 and DPSC1 on MG. Representative images (10X) were taken at 10, 13, 17, 20, and 24 dpn (*n* = 3). Scale bar represents 100 μm

To confirm that BDL-E5 specifically stains authentic reprogramming cells that eventually form colonies, ASCs and DPSCs were reprogrammed using the same episomal, feeder-free method. BDL-E5 staining was performed as described previously on the reprogrammed cells every 48 h, and images were taken from the same field of view daily until iPS colonies formed. Figure 3 shows representative images at 10, 13, 17, 20, and 24 dpn for reprogrammed ASCs and DPSCs. It is clear that only cells staining positive for BDL-E5 formed iPS colonies.

### BDL-E5^+^ reprogrammed cells generate higher quantity and quality of iPS cells

**Fig. 4:**
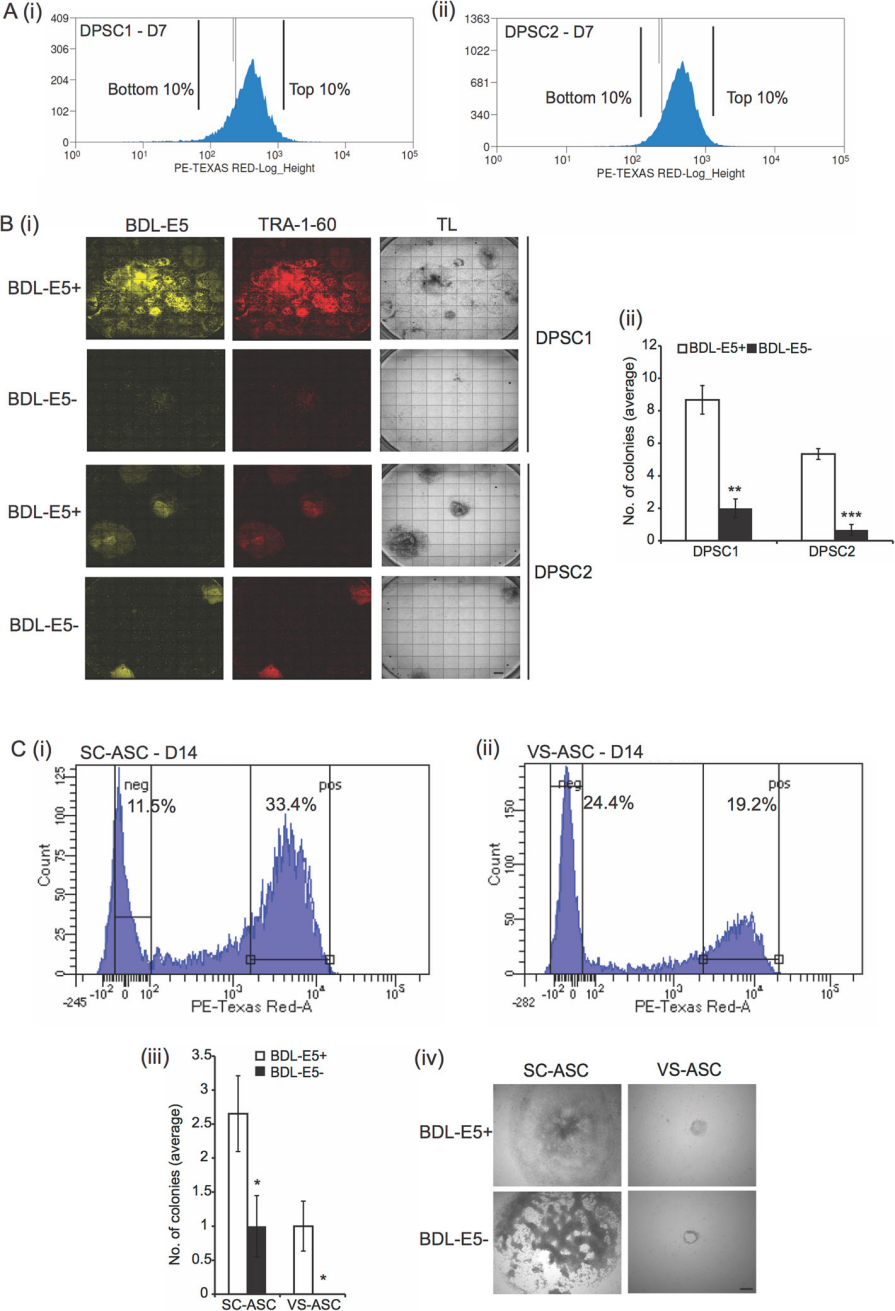
**a** Histogram (FACS) showing BDL-E5^+^ cell populations at 7 dpn: DPSC1 (i) and DPSC2 (ii). Relative cell count is indicated on the *y*-axis, and fluorescence intensity (Texas Red channel) on the *x*-axis. The top 10% and bottom 10% of cell populations are as indicated (*n* = 3). **b** (i) Fluorescent images of BDL-E5, TRA-1-60, and transmitted light (TL) images (4X) showing iPS colonies derived from BDL-E5^+^ and BDL-E5^−^ cell populations of DPSC1 and DPSC2 following FACS at 7 dpn. (ii) Graph showing average number of DiPS colonies in reprogrammed BDL-E5^+^ and BDL-E5^−^ cell populations obtained after FACS of DPSC1 and DPSC2. **p < 0.01 and ***p < 0.001 denote statistical significance (*n* = 3). **c** Histogram showing FACS of BDL-E5^+^ and BDL-E5^−^ cell populations at 14 dpn from SC-ASC S15 (i) and VS-ASC S15 (ii). The percentage of positively and negatively stained cells is shown. (iii) Graph showing average number of iPS colonies in BDL-E5^+^ and BDL-E5^−^ cell populations obtained after FACS at 14 dpn from SC-ASC S15 and VS-ASC S15. *p < 0.05 denotes statistical significance. (iv) TL images (10X) showing iPS colonies from SC-ASC S15 and VS-ASC S15 following FACS for BDL-E5^+^ and BDL-E5^−^ cell populations. Similar results were obtained with S16-derived SC-ASC and VS-ASC (*n* = 3). Scale bar represents 100 μm

Different DPSC cell lines were reprogrammed using the feeder-free episomal method, incubated with BDL-E5, and subjected to fluorescence activated cell sorting (FACS) at 7 dpn. As shown in Fig. 4a, the bottom 10% and top 10% of cells stained with BDL-E5 were sorted, collected, and seeded onto MEF-coated plates. Figure S3A (i) and (ii) represents the unstained DPSCs. The cells were allowed to grow for the next 2 weeks until colonies appeared. BDL-E5^+^ (top 10%, positively stained) cells gave rise to an increased number of iPSCs, while BDL-E5^−^ (bottom 10%, negatively stained) cells gave rise to significantly fewer colonies per well (Fig. 4b).

Next, we investigated whether the probe was useful in assisting reprogramming selection of obese patient-derived ASCs from subcutaneous (SC) and visceral (VS) fat depots. Unlike SC-derived ASCs, VS-derived ASCs exhibit cellular defects, including adipogenesis [18, 19]. It was found that VS-ASCs also showed substantial defects in reprogramming, typically resulting in < 1 colony being formed per well. Interestingly, when ASCs were subjected to FACS with BDL-E5 at 14 dpn, BDL-E5^+^, and BDL-E5^−^ populations of cells were more demarcated; SC-ASCs showed higher percentage of cells (~ 33%) staining positively for BDL-E5 and ~ 11% of cells negatively for BDL-E5 (Fig. 4c(i)). VS-ASCs showed a decreased proportion of cells staining positively (~ 19%) for BDL-E5 and an increased percentage (~ 24%) of negative cells, as shown in Fig. 4c(ii). Figure S3A (iii) and (iv) represents the unstained ASCs. BDL-E5^+^ and BDL-E5^−^ cells sorted using FACS were seeded onto MEF-coated plates. Quantification of the number of iPS colonies formed after plating clearly indicated that BDL-E5^+^ SC-ASCs gave rise to more colonies than the BDL-E5^−^ population (Fig. 4c(iii) and (iv)). Significantly, at least some BDL-E5^+^ VS-ASCs gave rise to iPS colonies whereas BDL-E5^−^ VS-ASCs did not (Fig. 4c(iii) and (iv)). Thus, these results demonstrate that BDL-E5 staining helps identify the cell population amenable to reprogramming and increases the chance of generating iPS colonies from difficult-to-reprogram cell types such as VS-ASCs.

To investigate the quality of the BDL-E5^+^ generated iPS cells, the iPS colonies were passaged for several generations. As shown in Fig. S3B(i), ASC-derived BDL-E5^+^ colonies remained well self-renewed and TRA-1-60 positive for subsequent passages. However, BDL-E5^−^ cells generated an average of only one colony, stained negative with TRA-1-60, and failed to form colonies upon subsequent passages (Figs. S3B(ii) and S3C).

### BDL-E5^+^ cells have increased expression of pluripotency and epithelial markers

**Fig. 5:**
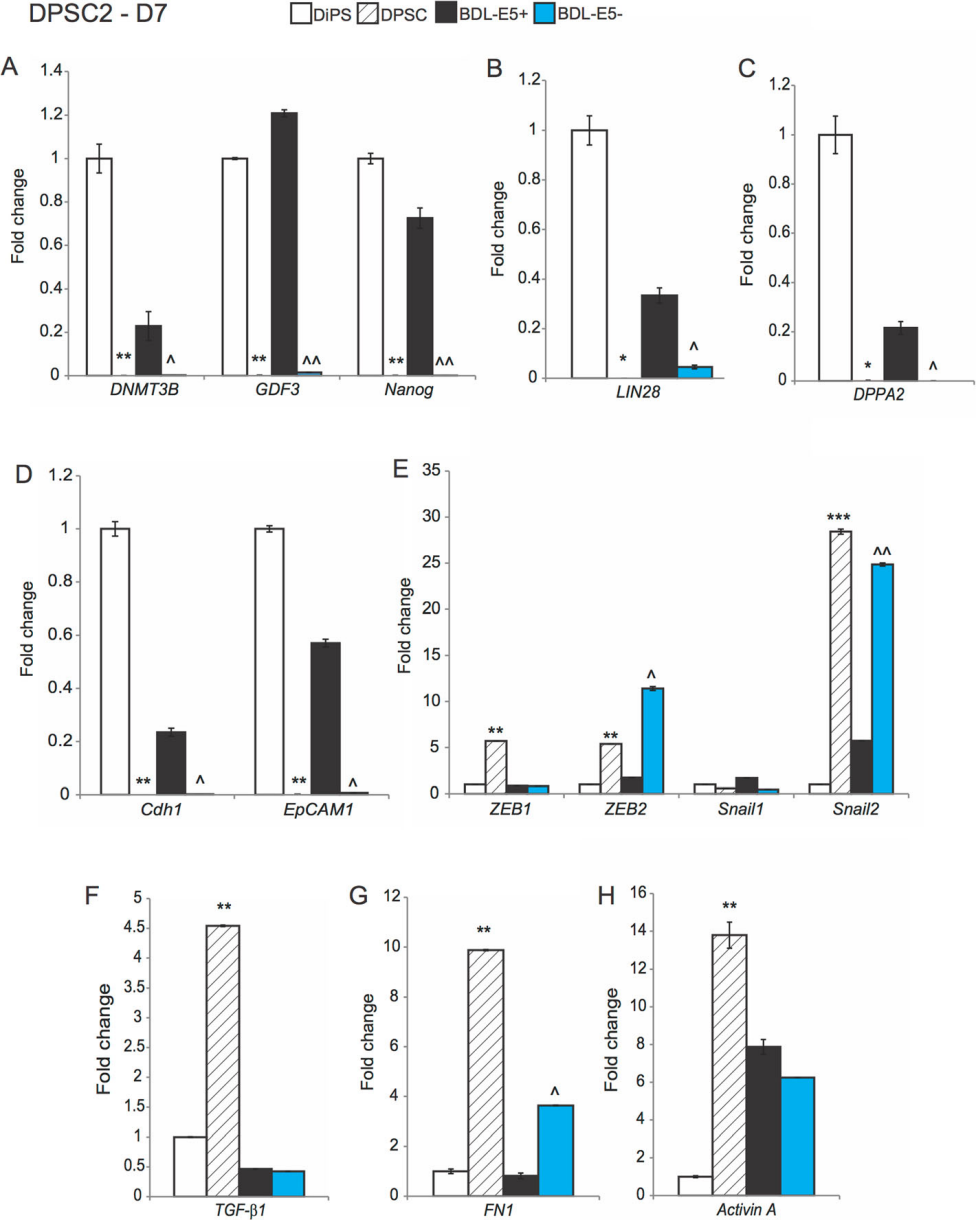
Representative graphs showing gene expression of *DNMT3B*, *GDF3*, and *Nanog* (**a**); *LIN28* (**b**); *DPPA2* (**c**); *Cdh1* and *EpCAM1* (**d**); *ZEB1*, *ZEB2*, *Snail1*, and *Snail2* (**e**); *TGF-β1* (**f**), *FN1* (**g**); and *Activin A* (**h**) in RNA isolated from DiPS2, DPSC2, BDL-E5^+^, and BDL-E5^−^ cells of DPSC2 obtained after FACS at 7 dpn (*n* = 3). DiPS2 colonies were generated from the original DPSC2 cells that were similarly subjected to FACS. *p < 0.05 and **p < 0.01 denote significance compared with DiPS2; ^*p* < 0.05 and ^^*p* < 0.01 denote significance compared with BDL-E5^+^

The process of cellular reprogramming from somatic to iPS cells involves mesenchymal-epithelial transition (MET) and increased expression of pluripotency genes [20, 21]. DPSCs were reprogrammed and FACS was performed with BDL-E5 at 7 dpn. Expression of pluripotent, epithelial, and mesenchymal genes was measured using qPCR in BDL-E5^+^ (top 10%) and BDL-E5^−^ (bottom 10%) cells. DiPS (dissociated into single cells) and DPSCs (non-reprogrammed) populations were also sorted by FACS and collected as positive and negative controls, respectively. As shown in Fig. 5a–d, BDL-E5^+^ cells exhibited increased expression of pluripotency genes, *DNMT3B*, *GDF3*, *Nanog*, *LIN28*, and *DPPA2*, and epithelial genes, *Cdh1* and *EpCAM1*, compared with BDL-E5^−^ cells. The increased expression of these genes in BDL-E5^+^ cells was comparable to that of DiPS cells, and decreased expression of these genes in BDL-E5^−^ cells was comparable to that of DPSCs. qPCR was used to measure expression of mesenchymal genes, *ZEB1*, *ZEB2*, *Snail1*, *Snail2*, *TGF-β1*, *FN1*, and *Activin A* (Fig. 5e–h). BDL-E5^+^ cells showed decreased expression of *ZEB2*, *Snail2*, and *FN1* compared with BDL-E5^−^ cells. BDL-E5^−^ cells showed increased expression of mesenchymal genes comparable to DPSCs. Gene expression measurements using qPCR in reprogrammed ASCs sorted with BDL-E5 at 14 dpn showed increased *LIN28* and *Nanog* expression in BDL-E5^+^ cells, while BDL-E5^−^ cells showed significantly increased expression of *TGF-β1* and slightly increased expression of *Activin A*, with a trend similar to AiPS versus ASCs (Fig. S4A).

To confirm that BDL-E5^+^-derived iPS cells are bona fide pluripotent cells, we generated embyoid bodies and allowed the cells to spontaneously differentiate in vitro. The differentiated cells were then subjected to three-germ layer immunohistochemistry. As shown in Fig. S4B(i), the differentiated cells exhibited positive staining for all three germ layers including ectodermal TUJ1, mesodermal SMA, and endodermal AFP. The qPCR analysis also indicated that the spontaneously differentiated cells derived from BDL-E5^+^ iPS cells showed increased gene expression of ectodermal *GATA2*, mesodermal *SMA*, and endodermal *AFP* and *SOX7* (Fig. S4B(ii)–(v)). Hence, these results supported authenticity of the BDL-E5^+^-derived iPS cells.

### BDL-E5 detects iPS cells generated with common protocols or three-dimensional (3D) culture conditions and may localize to the Golgi complex

In order to test whether BDL-E5 would be useful for identifying reprogramming and iPS cells in 3D culture suitable for large scale production, ASCs and DPSCs were reprogrammed and seeded on Geltrex-coated Cytodex 3 microcarriers prior to staining with BDL-E5 and TRA-1-60. As shown in Fig. S4C, BDL-E5 staining was clearly observed in reprogrammed cells in both cell lines, and pluripotency of the iPS cells formed on the microcarriers was confirmed using TRA-1-60 staining. The Cytodex 3 microcarriers themselves had no background BDL-E5 staining, and the reprogrammed cells were readily distinguishable with intense fluorescence, at both 14 and 21 dpn.

To determine the subcellular organelle localization of BDL-E5 in reprogramming cells, 7 dpn DPSCs on MG were stained for BDL-E5 and organelle marker dyes for endoplasmic reticulum (ER), Golgi complex, lysosome, or mitochondria. Confocal images from three independent experiments showed that BDL-E5 staining appeared to co-localize more significantly with the Golgi complex marker than with other organelle markers (Fig. S4D).

We also tested whether BDL-E5 worked for commonly used reprogramming methods and cell type. DPSCs were infected with retroviral vectors expressing four Yamanaka factors and plated onto MEF. BDL-E5 similarly stained reprogramming cells as early as 7 days post-infection (dpi), when TRA-1-60 failed to detect any cells (Fig. S5A). In addition, human BJ fibroblasts were reprogrammed with lentiviral Yamanaka factors. BDL-E5 successfully stained reprogramming, but not non-reprogramming cells, and staining was stronger than that of TRA-1-60, indicating that BDL-E5 detected reprogramming of different cell lines (Fig. S5B and C).

### RNA-sequencing analysis reveals early reprogramming markers in BDL-E5^+^ cells

**Fig. 6:**
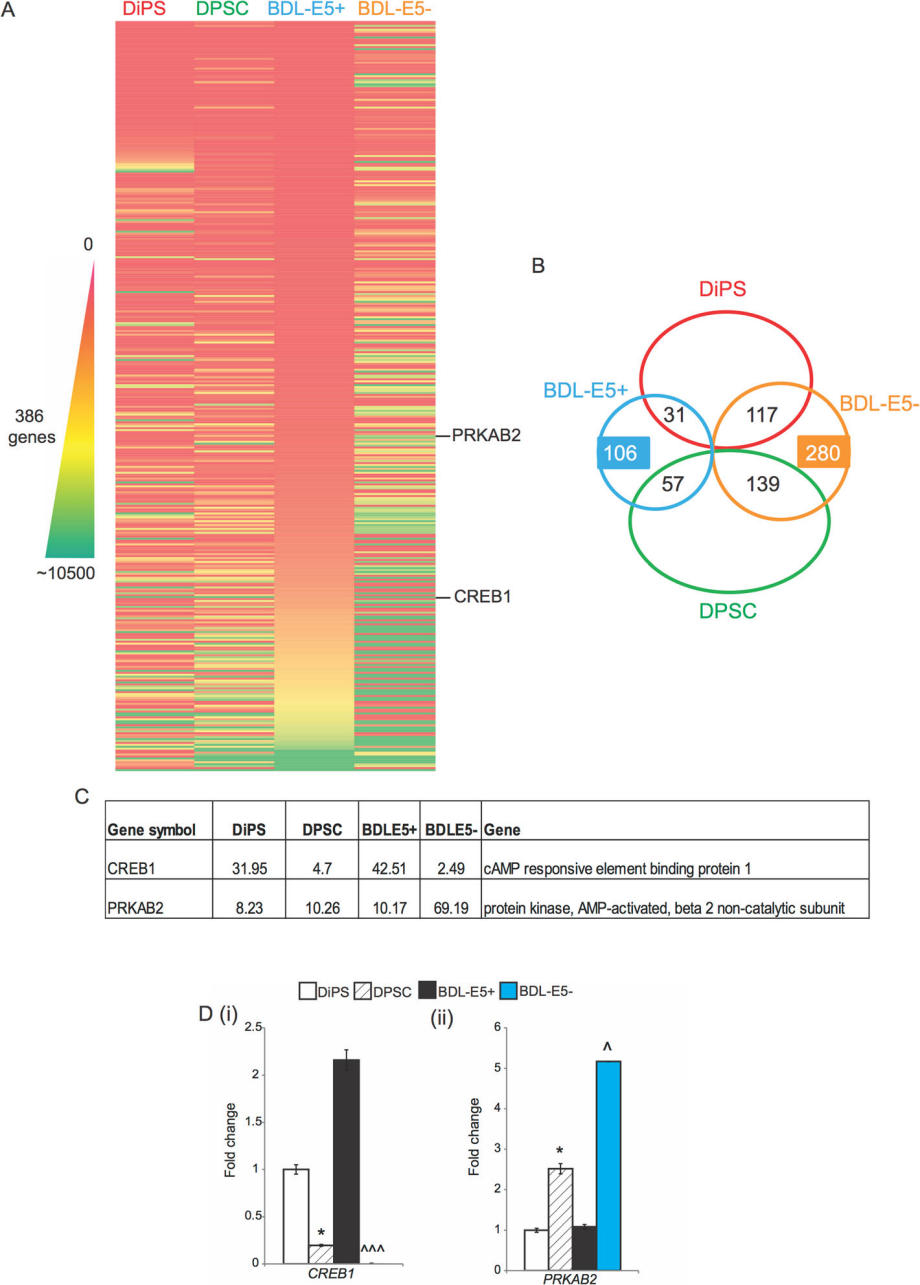
**a** Heatmap showing 386 differentially expressed genes (between BDL-E5^+^ and BDL-E5^−^ cells) after RNA sequencing of four cell types (DiPS, DPSC, BDL-E5^+^, and BDL-E5^−^ cells) after FACS at 7 dpn of DPSC2 upon reprogramming (*n* = 2). **b** Venn diagram showing the number of genes up-regulated in BDL-E5^+^ and BDL-E5^−^ cells, as obtained from RNA sequencing data. A total of 386 genes were differentially expressed significantly between the two cell types. **c** Table showing the relevant genes that were differentially expressed among DiPS, DPSC, BDL-E5^+^, and BDL-E5^−^ cells. **d** Graphs showing mRNA expression of *CREB1* (i) and *PRKAB2* (ii) obtained by qPCR from RNA isolated from DiPS, DPSC, BDL-E5^+^, and BDL-E5^−^ cells of DPSC2 obtained after FACS at 7 dpn (*n* = 3). *p < 0.05 denotes significance compared with DiPS; ^*p* < 0.05 and ^^^*p* < 0.001 denote significance compared with BDL-E5^+^

To identify classes of novel genes that might be involved in early reprogramming stages defined by BDL-E5, RNA sequencing was performed on BDL-E5^+^ and BDL-E5^−^ DPSCs sorted at 7 dpn, using DPSCs and DiPS cells as reference controls. Genes showing statistical significance (*p* < 0.05) and > 2 fold change were selected for analysis and, overall, 386 genes (shown in Table S1) were significantly differentially expressed (106 upregulated and 280 downregulated) in BDL-E5^+^ versus BDL-E5^−^ sorted cells as shown in a heatmap and Venn diagram (Fig. 6a, b). Further analysis of the 386 differentially expressed genes was carried out. Among the BDL-E5^+^ upregulated genes, 31 genes were expressed higher and 57 genes were expressed lower in DiPS cells compared with DPSCs. On the other hand, 117 genes were expressed higher and 139 genes were expressed lower in DiPS cells than DPSCs among the BDL-E5^+^ downregulated genes. Annotation using ingenuity pathway indicated that differentially regulated genes in BDL-E5^+^ versus BDL-E5^−^ cells were associated with “embryonic development,” “organismal development,” and “tissue development” categories. Top canonical pathway and molecular cellular functions included “BMP signaling pathway,” “FGF signaling,” “cell-to-cell signaling and interaction,” “cellular assembly and organization,” and “cellular growth and proliferation” (Fig. S6A). Another analysis was performed using Metascape and demonstrated that the top enriched clusters between BDL-E5^+^ and BDL-E5^−^ cells included “lamellipodium morphogenesis,” “positive regulation of organelle organization,” “regulation of transporter activity,” “cell morphogenesis involved in neuron differentiation,” and “embryo development” (Fig. S6B).

Among these, we were particularly interested in *CREB1* and *PRKAB2* genes due to their potential involvement in the metabolic reprogramming process (Fig. 6a, c). Expression of *CREB1* was significantly upregulated in BDL-E5^+^ cells compared with BDL-E5^−^ cells. *CREB1* was also upregulated in DiPS cells. *PRKAB2* was downregulated in both DiPS and BDL-E5^+^ cells. Gene expression was further confirmed by qPCR as shown in Fig. 6d. Based on these results, we hypothesized that the pathway regulated by CREB1 may be involved in the early reprogramming process of cells that are marked by BDL-E5.

### CREB1 affects reprogramming efficiency

**Fig. 7:**
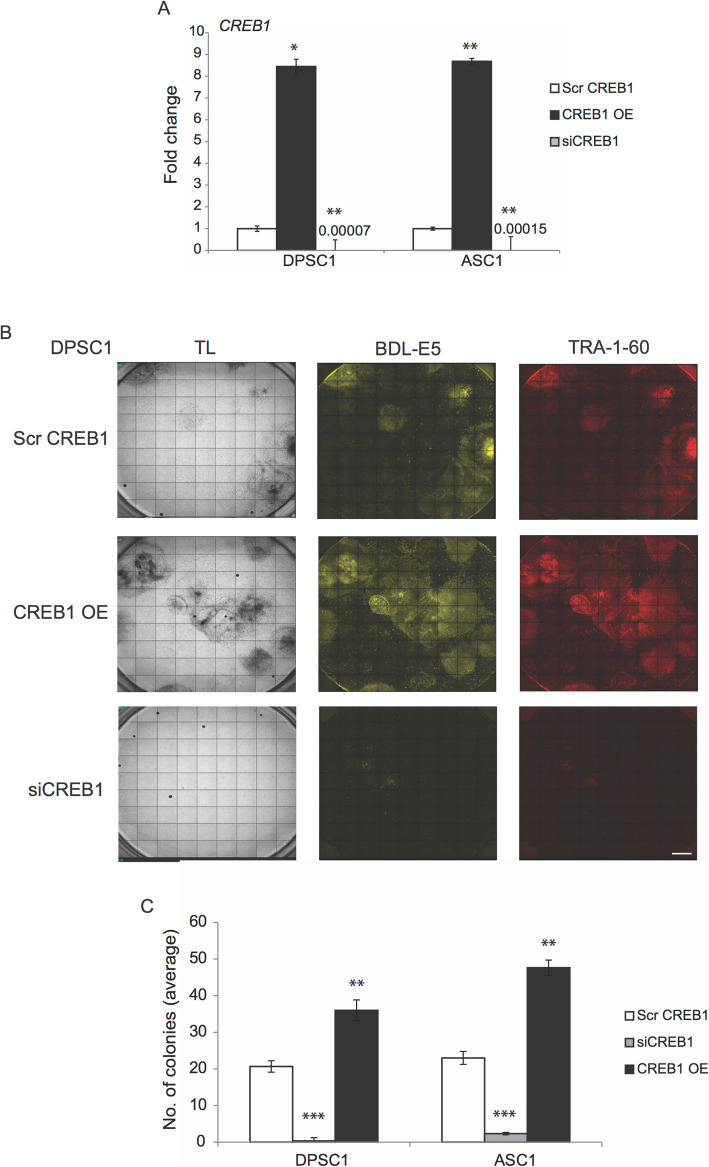
**a** Graph showing mRNA expression of *CREB1* in Scr CREB1, CREB1 OE, and siCREB1 DPSC1 and ASC1 (*n* = 3). *p < 0.05 and **p < 0.01 denote significance compared with Scr DPSC1/ASC1. **b** TL and fluorescence images (10X) of BDL-E5, TRA-1-60 showing iPS colonies derived from DPSC1 transfected with Scr CREB1, CREB1 OE, and siCREB1 at 12 dpn (*n* = 3). Scale bar represents 100 μm. **c** Graph showing the average number of colonies in reprogrammed DPSC1 and ASC1 transfected with Scr CREB1, CREB1 OE, and siCREB1 at 12 dpn (*n* = 3). **p < 0.01 and ***p < 0.001 denote statistical significance

In order to determine whether CREB1 plays a role in the reprogramming process and thus affects reprogramming efficiency, overexpression and knockdown of CREB1 were performed using *CREB1* overexpression (CREB1 OE) vectors and siRNA targeting *CREB1* (siCREB1), respectively. DPSC1 and ASC1 were either nucleofected with the CREB1 OE vectors or transfected with siCREB1 during reprogramming, then nucleofected with the episomal reprogramming factors. mRNA expression of *CREB1* was significantly increased with *CREB1* overexpression and significantly decreased with *CREB1* knockdown in both DPSC and ASC lines (Fig. 7a). As shown in Fig. 7b, c, overexpression of CREB1 increased the reprogramming efficiency in terms of the number of colonies and TRA-1-60-positive cells compared with the control (Scr CREB1) cells. Interestingly, knockdown of *CREB1* drastically decreased the reprogramming efficiency and on average only < 1 iPS colony per well was generated (Fig. 7b, c). Knockdown and overexpression of *CREB1* in ASCs also showed similar results. Knockdown of *CREB1* significantly reduced the number of colonies and overexpression of *CREB1* significantly increased the number of colonies formed (Fig. 7c). These results showed that *CREB1* expression levels significantly influence reprogramming efficiency, indicating an important role of CREB1 in the reprogramming process into induced pluripotency.

## Discussion

While iPS cells offer a broad range of potential applications including human disease modeling, personalized therapeutic screening, and ultimately, regenerative cell therapies, very few tools are available to readily identify emerging iPS cells early. This has hampered clinical development toward the prompt isolation of bona fide human iPS cells and our full understanding of the early process of iPS reprogramming. Traditionally, gene reporters such as fluorescent proteins driven under OCT4, NANOG, or artificial reporters have been used [22, 23]. However, these constructs need to be inserted into cells by viral, gene editing or other genetic engineering methods, and successful expression verified, which are cumbersome and potentially disruptive to endogenous genome function. Alternatively, several fluorescence-conjugated dyes based on alkaline phosphatase, SSEA3/4, or TRA-1-60/81 have been widely used. However, these probes stain already-visible iPS colonies are expensive to manufacture and are often not very specific to authentic pluripotent stem cells [9]. Some somatic cells such as ASCs and DPSCs were reported to be positive for SSEA4 and alkaline phosphatase activity. Here, we identified a cost-effective small molecule BODIPY-based fluorescent probe, BDL-E5, which specifically stains early reprogramming and mature iPS cells, but not ASCs or DPSCs. We and others have previously reported other fluorescent chemicals, CDy1 and KP-1 [12, 13, 24]. KP-1 is a rhodamine compound that was selected against human iPS cells; however, it suffers from limited specificity against pluripotent stem cells. Accordingly, when we attempted to sort early reprogramming cells, KP-1 failed to enrich high numbers and quality iPS colony formation [24]. CDy1, a rosamine derivative, was initially screened and identified against mouse ES and iPS cells. Whether CDy1 can sort and enrich reprogramming-ready population has not been investigated. Here, we confirmed use of CDy1 for identifying human iPS cells as well. When compared, CDy1 had higher signals toward human reprogramming cells in general; however, it also gave high background staining against non-reprogramming cells as demonstrated by signal-to-noise ratio measurement (Fig. S7A). In contrast, BDL-E5 exhibited low non-specific staining against non-reprogramming cells, and unlike CDy1, does not require washing after the staining process to reduce background signals. Furthermore, we performed proliferation assays and confirmed that BDL-E5 was not toxic to the cells (Fig. S7B). BDL-E5 specifically detected reprogramming of different cell lines including ASCs, DPSCs, and BJ fibroblasts.

There are several characteristics of BDL-E5 that make it potentially useful for clinical and commercial applications. BDL-E5 allows early detection of reprogramming cells, approximately 7 days before iPS colonies are visible, and stains the pluripotent cell surface marker TRA-1-60. BDL-E5 can be used to sort early reprogrammed cells and select cell populations that will later generate higher numbers and quality iPS colonies. We also showed that BDL-E5 can stain reprogrammed iPS cells that were 3D-cultured with microcarriers, indicating its potential applications as a quality control agent toward industrial manufacturing of iPS cells. BDL-E5 also allows iPS generation even from the difficult-to-reprogram cell type such as VS-ASCs. In addition, after differentiating iPS cells into desired cell types used for cell therapies (e.g., neurons, cardiomyocytes, hepatocytes), it is necessary to remove undifferentiated iPS cells. Otherwise, a single remaining iPS cell may be sufficient to form teratoma after transplantation into patients. BDL-E5 allows easy detection of undifferentiated iPS cells, making it easier to deplete such unwanted cells in the final stage of clinical production.

Selection of early reprogrammed cell populations with BDL-E5 and subsequent gene expression analysis revealed sets of previously recognized as well as unrecognized genes that are involved in the early reprogramming process toward induced pluripotency. qPCR analysis indicated that BDL-E5^+^ reprogrammed cells exhibit high expression levels of pluripotent genes, some of which are nearly comparable to those in mature iPS cells. It was demonstrated that the early stage of reprogramming requires MET [20, 21]. Consistently, BDL-E5^+^ reprogrammed cells were characterized by downregulation of several mesenchymal genes and upregulation of representative epithelial genes. In addition, RNA-seq analysis of BDL-E5^+^ and BDL-E5^−^ cells along with their original somatic and resultant iPS control cells showed a further novel set of genes (Figs. 6 and S6). Among the genes that stood out was CREB1, a transcription factor that controls a number of downstream target genes that harbor cAMP response element (CRE). While CREB1 has been shown to be important for differentiation of pluripotent stem cells into neuronal cell types [25], its role in reprogramming of human pluripotent stem cells has not been reported. We found that CREB1 plays an important role in reprogramming into human iPS cells. Importantly, CREB1 overexpression or knockdown does not affect the proliferation of cells (Fig. S7C). Previous studies used forskolin, an adenylyl cyclase activator that increases intracellular cAMP level and activates CREB1, as part of a chemical cocktail to enhance iPS reprogramming, at least in mouse cells [26, 27]. Further studies are necessary to delineate the cAMP-dependent pathway and potential targets of CREB1 during reprogramming of human cells. Taken together, BDL-E5 is valuable for identifying early reprogramming cell populations and makes it possible to discover unexplored mechanisms of the human iPS reprogramming process.

## Conclusions

From the DOFLA screening, we identified a novel BODIPY-based fluorescent probe, BDL-E5, which specifically detects human iPS cells at the early reprogramming stage. The dye is specific to human iPS cells, and not to their original somatic cells, where some pluripotent markers are expressed. BDL-E5 detected pluripotent cells around 7 days before iPS colonies were visible and stained with the conventional antibody-based pluripotent marker Tra-1-60. Cell sorting with BDL-E5 enriches cell population amenable to forming iPS colonies and allows iPS generation even from the difficult-to-reprogram cell type. The dye can be used for microcarrier-based 3D culture system, which would be useful for the commercial scale of iPS production. Comprehensive gene expression analysis by RNA sequencing highlighted characteristic early reprogramming gene signatures, which included CREB1 and other factors. We subsequently found that CREB1 plays a direct role in the early reprogramming process into iPS cells. Collectively, our new fluorescent tool has significant implication in scientific and translational applications of human iPS cells.

## Materials and methods

### Isolation of ASCs

WAT was isolated from subcutaneous (abdominal region) and visceral (omental region) depots from 2 human volunteers (S15-S16, undergoing bariatric surgery, with approval by the National Healthcare Group Domain Specific Review Board at National Healthcare Group, Singapore) as described previously [18, 19]. S15 is a 20–29 year old Indian 1 and S16 is a 30–39-year-old Indian 2. ASCs were isolated from WAT and cultured, as previously described [4]. Cells only up to passage 5 were used for experiments. MSC cell surface markers and multipotency of ASCs used in this study were confirmed by flow cytometry and differentiation assays, respectively [18].

### ASC and DPSC culture

Different lines of ASCs and DPSCs were obtained from commercial sources (Lonza, Invitrogen, and PromoCell). ASCs were cultured in DMEM containing 15% FBS, NEAA (1%), basic FGF (bFGF; 5 ng/ml), and Pen/Strep as previously described [4, 18, 19], and DPSCs were grown in vitro in Poietics™ DPSC BulletKit medium (Lonza) according to manufacturer’s instructions. Media change for the cells was performed every 2–3 days. All cells were cultured in a humidified incubator at 37 °C in 5% CO_2_.

### iPS reprogramming using episomal vectors

Episomal plasmids developed by Yamanaka’s lab were obtained from Addgene: pCXLEhOct3/4-shp53-F (Addgene # 27077), pCXLE-hSK (Addgene # 27078), pCXLE-hUL (Addgene # 27080), and pCXLE-EGFP (Addgene # 27082) [28]. 1 × 10^6^ cells were suspended together with 1 μg of each episomal vector in Nucleofector solution supplied in the Nucleofector Kit R (Lonza). Then, the cells were transfected with the Program FF-113 on a Nucleofector 2b Device. The transfected cells were then cultured in ASC or DPSC medium (MSC medium) supplemented with 0.5 mM sodium butyrate, with daily media change. On day 7 dpn, 1 × 10^5^ viable cells were seeded over MEF feeders (GlobalStem) into one well of a 6-well plate for feeder-based iPS derivation; 2 × 10^5^ viable cells were seeded for feeder-free iPS derivation into one well of a 6-well plate pre-coated with Matrigel (Corning). The next day, MSC medium was changed to feeder-based hES medium (DMEM/F12 supplemented with 20% knock out serum replacement, 1% GlutaMAX, 1% NEAA, Pen/Strep, 0.1 mM β-mercaptoethanol, and 10 ng/ml b-FGF) or to feeder-free mTeSR1 (StemCell Technologies), supplemented with sodium butyrate. At 12 dpn, supplementation of sodium butyrate was stopped and conditioned further with SMC4 cocktail (consisting of small molecules: PD0325901, CHIR99021, Thiazovivin, and SB431542 (FOCUS Biomolecules)) in hES medium/mTeSR1. This media supplement was continued until initial colony formation began.

### Fluorescent probes and screening

The chemical properties of the BDL library are previously described [29]. BDL-E5 is based on 4,4-difluoro-4-bora-3a,4a-diaza-s-indacene (BODIPY), with calculated mass of 528.3 and absorption maximum/emission maximum of 578/599 nm. Primary, secondary, and tertiary screening of fluorescent probes on AiPS, ASCs, DiPS, and DPSCs were performed as described previously [12, 13] and in the “Results” section. Unless described otherwise, BDL-E5 and CDy1 images were acquired by the tetramethylrhodamine (TRITC) channel of ImageXpress Micro High-Content Imaging System, which had the adaptive background correction function enabled. Semi-automated, non-discriminatory quantification of BDL-E5 and other staining images were performed as previously described.

### Immunofluorescence live cell staining

Reprogrammed cells were immune-stained with fluorescent live cell stain TRA-1-60 (R&D Systems, GloLIVE NL557) as per manufacturer’s instructions. After incubating with the live staining antibodies for 30 min and Hoechst 33342 for 10 min, cells were washed 3 times with PBS and images with the Cy5 and DAPI channels, respectively, were immediately captured.

### Fluorescent activated cell sorting (FACS)

Reprogrammed ASCs and DPSCs, AiPS and DiPS on D7 or D14 dpn were stained with BDL-E5 for 1 h and then harvested using TrypLE and resuspended in 1X PBS. The cells were then subjected to FACS in the MoFlo XDP Cell Sorter (Beckman Coulter) under sterile conditions. The sorted cells were either collected for RNA sequencing, real-time PCR, or re-seeded onto MEF-coated plates at a density of 10,000 cells/cm^2^.

### RNA sequencing

At 7 dpn, reprogrammed DPSC2 cells were stained with BDL-E5 and harvested for FACS as mentioned above. Twenty to 30 BDL-E5^+^ and BDL-E5^−^ cells were collected in 100 μl of 1X PBS; single cells of DiPS and DPSCs were also passed through the Cell Sorter and collected for RNA isolation. RNA was isolated from single cells and cDNA preparation, amplification, and quantification were as described in the “Supplementary Methods” section. Library preparation and sequencing were done by sequencing platform at Genome Institute of Singapore. Paired-end RNA sequencing reads were aligned to the human genome (hg19) using TopHat2-2.0.12 [30] (default parameter). Transcript abundances at both the gene and isoform levels were estimated by cufflinks-2.2.0 [31], and the expression was reported as fragments per kilobase of exon per million fragments mapped (FPKM).

### Real-time PCR

Total RNA was extracted using TRIzol reagent (Invitrogen), and cDNA conversion was made by the RevertAid H minus the first-strand cDNA synthesis kit (Fermentas) as per manufacturer’s instructions. qPCR was performed using SYBR Green PCR Master Mix on a StepOnePlus Real-Time PCR System (Applied Biosystems) using the primer pairs shown in Table S2. Relative mRNA was calculated and normalized to the level of *GAPDH*.

### CREB1 overexpression and silencing by siRNA

CREB1 was overexpressed in ASCs and DPSCs during reprogramming using the commercially available CREB1 Human cDNA ORF clone (Origene) according to manufacturer’s instructions. Knockdown of CREB1 was achieved using the ON-TARGETplus Human CREB1 siRNA – SmartPool (GE Dharmacon) according to manufacturer’s instructions. DPSCs and ASCs were either nucleofected with the CREB1 OE along with the episomal reprogramming factors for overexpression of *CREB1* during reprogramming or transfected with siCREB1 and then nucleofected with the episomal reprogramming factors for silencing of *CREB1* during reprogramming.

### Statistical analysis

All results are presented as means +/− SEM. Statistical analysis was performed using *t* tests (two sided; paired). Differences with *p* value < 0.05 were considered significant.

## Supplementary Information

**Additional file 1.** Supplemental methods.

**Additional file 2: Figure S1.** (A) Fluorescent images (10X objective) of CDy1 probe (Wash 180 min) and Hoechst on AiPS1 colonies and ASC1 on (i) MEF- and (ii) MG-coated plates from primary screening (*n* = 3). (B) Fluorescent images (10X) of CDy1 probe (Wash 180 min), Hoechst and TRA-1-60 on DiPS1 colonies and DPSC1 on MEF- and MG-coated plates from secondary screening. Cells were incubated with 500 nM of CDy1 in appropriate media for 1 h (n = 3). Scale bar represents 100 μm.

**Additional file 3: Figure S2.** (A) Fluorescent images (10X) of BDL-E5, CDy1 and Hoechst on AiPS1 colonies on MEF- (A) and MG-coated (B) plates at different conditions (No wash, Wash 0 min, Wash 60 min, Wash 180 min) after incubation with 500 nM probe for 1 h (*n* = 3). *Represents the same images that are presented in Figs. 2 and S1. (C) Fluorescent images (10X) of BDL-E5 and CDy1 on AiPS3 colonies on MEF- (A) and MG-coated (B) plates at different conditions (No wash, Wash 0 min, Wash 60 min) after incubation with 500 nM probe for 1 h (*n* = 3). Scale bar represents 100 μm.

**Additional file 4 **: **Figure S3.** (A) (i)-(iv) Histogram (FACS) showing unstained populations of cells used as the control for FACS performed in Fig. 4. (B) Fluorescence images of BDL-E5, TRA-1-60 and transmitted light (TL) images showing iPS colonies derived from ASC4 14 dpn BDL-E5^+^ (i) and BDL-E5^−^ (ii) cells at passage 0 (4X) and passage 4 (10X) (*n* = 3). Scale bar represents 100 μm. (C) Graph showing average number of iPS colonies from BDL-E5^+^ and BDL-E5^−^ cell populations at 14 dpn in ASC4 at passage 0 (n = 3). ***p < 0.001 denotes statistical significance.

**Additional file 5: Figure S4.** (A) Representative graphs showing gene expression of *LIN28* (i), *NANOG* (ii), *Activin A* (iii) and *TGF-β1* (iv) in RNA isolated from AiPS4, ASC4, BDL-E5^+^ and BDL-E5^−^ cells of ASC4 at 14 dpn. *p < 0.05 and ***p < 0.001 denote significance compared with AiPS4; ^^*p* < 0.01 denotes significance compared with BDL-E5^+^ (n = 3). (B) (i) Fluorescence images (10X) of TUJ1, SMA, AFP, DAPI and TL of cells following spontaneous differentiation of EBs generated from BDL-E5^+^ DPSC1. (ii)-(v) Representative graphs showing gene expression of *GATA2, SMA, AFP* and *SOX7* in RNA isolated from DiPS1 and spontaneously differentiated cells from EBs formed from BDL-E5^+^ iPS cells. ***p < 0.001 and ****p < 0.0001 denote significance compared with DiPS1 (n = 3). (C) Phase contrast (PC) and fluorescent images of BDL-E5 and TRA-1-60 of reprogramming SC-ASC S16 and DPSC2 on Geltrex™-coated Cytodex 3 microcarriers at 14 dpn (10X) and 21 dpn (20X). Scale bar represents 100 μm. (D) Fluorescent images of reprogramming DPSC2 on MG coated chamber slides at 7 dpn (n = 3). These images are zoomed in and cropped from 20X images to clearly show the stains and their overlap; green – markers for Endoplasmic Reticulum (ER), Golgi, Lysosome, or Mitochondria; red – BDL-E5; blue – Hoechst 33342.

**Additional file 6:.** **Figure S5.** (A) DPSC1 was reprogrammed with the traditional method involving retroviral OCT4, SOX2, KLF4 and C-MYC, and plated onto the MEF feeder layer. Cells were co-stained with BDL-E5 (yellow), TRA-1-60 (red) and Hoechst 33342 (blue) in the indicated day post-infection (dpi). (B) BJ fibroblasts were transduced with lentiviral OCT4, SOX2, KLF4 and C-MYC in the presence or absence of A83–01 (0.3 μM) and stained at 8 dpi. The image is merged from 9 independent fields. (C) BJ fibroblasts transduced above were stained with BDL-E5 followed by cell fixation and immunostaining with TRA-1-60 at 21 dpi.

**Additional file 7: Figure S6.** (A) Pathway analysis using Ingenuity Systems (Qiagen) shows representation of the top networks and canonical pathways between BDL-E5^+^ and BDL-E5^−^ cells. The molecular and cellular functions that were differentially expressed in BDL-E5^+^ and BDL-E5^−^ cells are also represented, along with the *p* values. (B) Metascape gene analysis was performed on http://metascape.org and the enriched clusters between BDL-E5^+^ vs. BDL-E5^−^ cells are represented here.

**Additional file 8: Figure S7.** (A) Graph representing signal-to-noise ratios (arbitrary fluorescence units) on comparing reprogramming (RP) versus non-reprogramming (non-RP) DPSCs (DPSC1) stained with either CDy1 or BDL-E5. The fluorescence intensity was measured using ImageJ software. 100 cells per field (10X), 10 fields per well, 3 wells per probe were measured. ****p < 0001 denotes significance between RP and non-RP cells. (B) Proliferation assay of DPSC1 incubated with BDL-E5 (500 nM) for 2 to 5 days; represented as number of viable cells per cm^2^ (*n* = 3). (C) Proliferation assay of reprogramming DPSC1, 48 h after transfection with Scr CREB1, CREB1 OE or siCREB1; represented as number of viable cells per cm^2^ (n = 3).

**Additional file 9:.** **Table S1.** List of the 386 genes differentially expressed in DiPS, DPSC, BDL-E5+, BDL-E5-.

**Additional file 10:.** **Table S2.** Real time qPCR primers.

## Data Availability

The datasets analyzed and materials used during the current study are available either commercially or from the corresponding author upon request.

## Abbreviations

AiPS: ASC-derived iPS
ASC: Adipose-derived stromal cell
BODIPY: 4,4-Difluoro-4-bora-3a,4a-diaza-s-indacene
CREB1: cAMP responsive element binding protein 1
DOFLA: Diversity Orientated Fluorescence Library Approach
dpn: Day post nucleofection
DiPS: DPSC-derived iPS
DPSC: Dental pulp-derived stem cell
ES: Embryonic stem
FACS: Fluorescence-activated cell sorting
iPS: Induced pluripotent stem
MEF: Mouse embryonic fibroblasts
MET: Mesenchymal-epithelial transition
MG: Matrigel
MSC: Mesenchymal stem cell
SC: Subcutaneous
VS: Visceral
