# Supplementary material for: Novel live cell fluorescent probe for human-induced pluripotent stem cells highlights early reprogramming population
Source: Stem Cell Res Ther. 2021 Feb 5;12:113. doi: 10.1186/s13287-021-02171-6 (PMC7866770; doi:10.1186/s13287-021-02171-6)
Supplement: Supplementary file 1 — Additional file 1. Supplemental methods. [file 13287_2021_2171_MOESM1_ESM.docx]

**Supplemental information for**

**Novel live cell fluorescent probe for human iPS cells highlights early reprogramming population**

Sandhya Sriram, Nam-Young Kang, Subha Subramanian, Tannistha Nandi, Samydurai Sudhagar, Qiaorui Xing, Gerine Jin-Ling Tong, Allen Kuan-Liang Chen, Thekkeparambil Chandrabose Srijaya, Patrick Tan, Yuin-Han Loh, Young-Tae Chang, Shigeki Sugii

**Supplemental methods**

**Generation of embryoid bodies and 3-germ layer immunocytochemistry**

For spontaneous *in vitro* differentiation, DPSC-derived iPS (DiPS) cells were grown to confluency. Using dispase, the cells were resuspended in medium containing DMEM-F12 supplemented with 10% Knockout Serum Replacement (KOSR), 1% [Non-Essential Amino Acid (NEAA)](http://www.cyagen.com/us/en/product/non-essential-amino-acid-cell-culture-supplement-100ml.html) and 1% Glutamax. These cells were transferred to low attachment 6-well plates (Greiner Bio One). Media change was made every 3 days. Embryoid bodies (EBs) were formed as previously described (1), day 8-10 EBs were transferred to a 12-well plate precoated with 0.1% gelatin and cultured further for 12 more days. Subsequently, the attached EBs were allowed to undergo spontaneous differentiation. These differentiated cells were later stained with 3 Germ Layer Immunocytochemistry antibodies (Life Technologies) as per the manufacturer’s instructions.

**Generating and culturing of reprogrammed cells on Cytodex 3 microcarriers**

Reprogrammed monolayer ASCs and DPSCs were dissociated into single cell suspension using Dispase. Single cell suspension was transferred into a 6-well Suspension Culture Plate (Greiner bio-one) with matrigel (Geltrex^TM^, ThermoFisher)-coated Cytodex 3 microcarrier in 4ml of mTeSR1 supplemented with 10 µM of Rock Inhibitor Y27632 (Calbiochem). The plate was placed on an orbital shaker (110 rpm) for at least 2 hours for cell attachment. Afterward, the plate was transferred to static condition and incubated at 37^o^C/5% CO_2_. Media change was carried out with mTeSR1 daily thereafter by aspirating 4ml of spent medium from the well and adding 4 ml of fresh media (80% medium exchange). On Day 7, the cells on microcarriers were supplemented and maintained with 4 ml of mTeSR1+SMC4 media. 80% medium change was carried out every day.

**Fluorescent probe staining on microcarriers**

BDL-E5 probe staining of reprogrammed ASCs and DPSCs on microcarriers was carried out on Day 14 and 21 dpn. The microcarrier culture was washed twice with sterile D-PBS. Prior to staining, media was changed to mTeSR1 with 500 nM BDL-E5 probe and Alexa Fluor^®^ 488 Mouse anti-human TRA-1-60 (200x dilution) (BD Pharmingen™). Cultures were incubated for 1 hour. The cells were then washed twice with D-PBS and replaced with mTeSR1 prior to imaging. Images were taken using Axio Observer fluorescent Microscope (Carl Zeiss).

**Fluorescent subcellular organelle staining**

Reprogrammed DPSCs at 7 dpn on MG were stained for cell organelle marker dyes (Molecular Probes) for endoplasmic reticulum (ER) (ER-Tracker™ Green (BODIPY® FL Glibenclamide)), Golgi complex (BODIPY® FL C5-Ceramide (N-(4,4-Difluoro-5,7-Dimethyl-4-Bora-3a,4a-Diaza-s-Indacene-3-Pentanoyl)Sphingosine)), lysosome (LysoTracker® Green DND-26), or mitochondria (MitoTracker® Green FM). Confocal images were taken to visualize the staining.

**iPS reprogramming using viral methods**

iPS cells were also generated from DPSCs with the traditional protocol involving retroviral vectors expressing OCT4, SOX2, KLF4, and C-MYC (2). BJ human neonatal fibroblasts were reprogrammed using lentiviral OCT4, SOX2, KLF4, and C-MYC as described previously (3).

**Single cell RNA isolation for RNA sequencing**

- Prepared Mix A (Lysis), Mix B (RT) and Mix C (PreAmp)


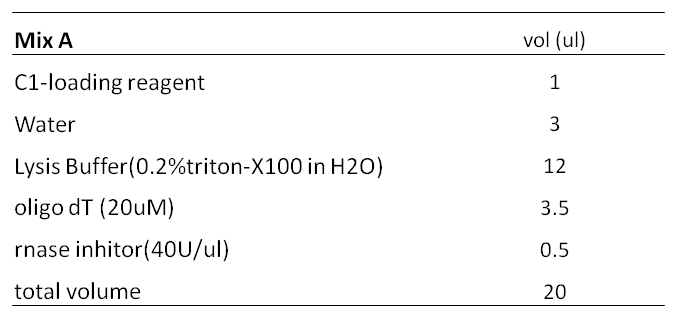


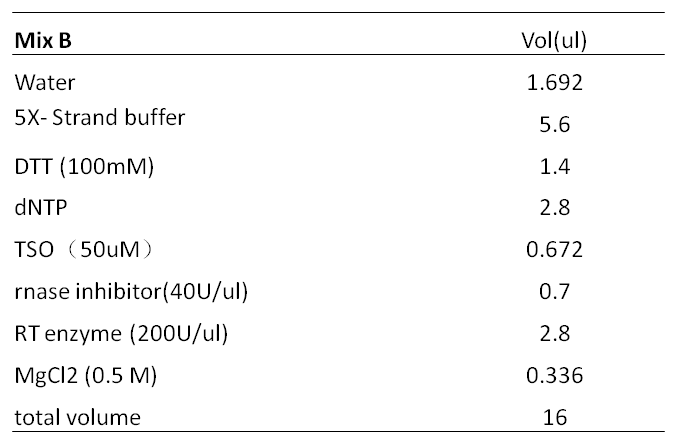


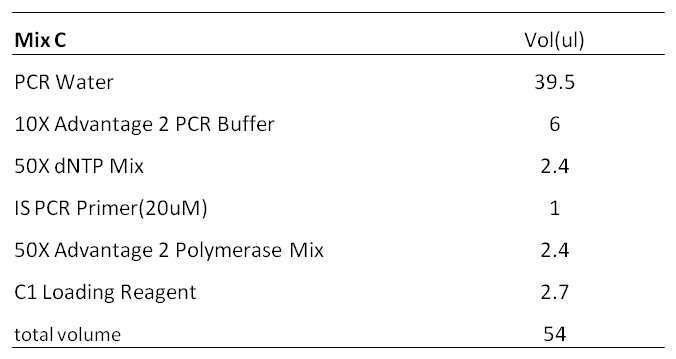


- Dissociated cells into single cell suspension
- Aliquoted 2 µl mix A into a 200ul thin PCR tube.
- Added 1 µl single cell suspension into the PCR tube as following.


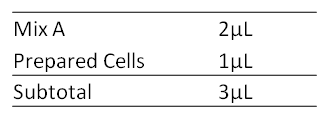


- The lysis step was run by the following program.


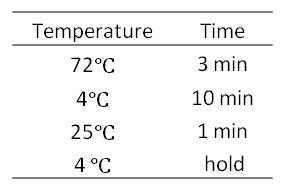


- Mix B was combined with lysis thermal products from step 4


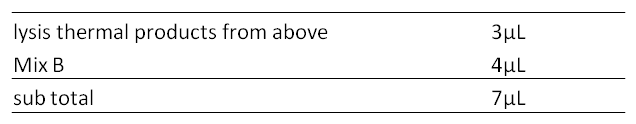


- Following program was run:


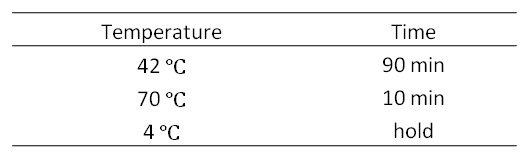


- Mix C was combined with RT thermal products from step 6


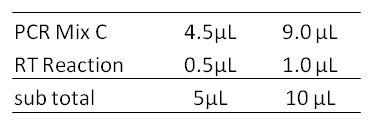


- Program was run as follows:


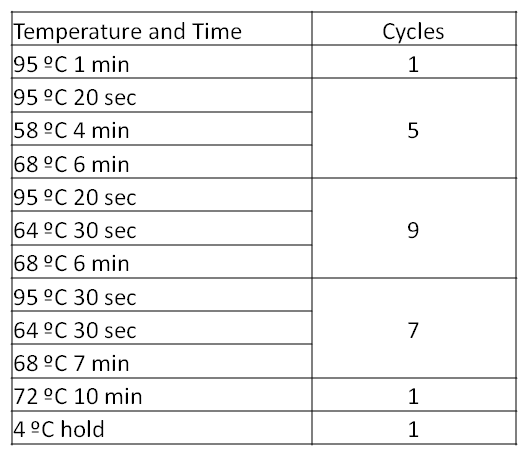


**Proliferation assay**

To ensure that BDL-E5 was not toxic to the cells, DPSCs were incubated with BDL-E5 (500 nM) for 48 h and 72 h and viable cells were counted in a haemocytometer using the Tryphan Blue method. DPSCs in which CREB1 was over-expressed or silenced were also counted at 72 h post nucleofection to determine if gene manipulation has affected the cell proliferation.

**Supplemental references**

1. Kurosawa H (2007) Methods for inducing embryoid body formation: in vitro differentiation system of embryonic stem cells. *J Biosci Bioeng* 103(5):389-398.

2. Sugii S, Kida Y, Berggren WT, & Evans RM (2011) Feeder-independent iPS cell derivation from human and mouse adipose stem cells. *Nature Protoc* 6(3):346-358.

3. Toh CX*, et al.* (2016) RNAi Reveals Phase-Specific Global Regulators of Human Somatic Cell Reprogramming. *Cell Rep* 15(12):2597-2607.

**Supplemental figure legends**

**Figure S1**

(A) Fluorescent images (10X objective) of CDy1 probe (Wash 180 min) and Hoechst on AiPS1 colonies and ASC1 on (i) MEF- and (ii) MG-coated plates from primary screening (n=3). (B) Fluorescent images (10X) of CDy1 probe (Wash 180 min), Hoechst and TRA-1-60 on DiPS1 colonies and DPSC1 on MEF- and MG-coated plates from secondary screening. Cells were incubated with 500 nM of CDy1 in appropriate media for 1 h (n=3). Scale bar represents 100 µm.

**Figure S2**

(A) Fluorescent images (10X) of BDL-E5, CDy1 and Hoechst on AiPS1 colonies on MEF- (A) and MG-coated (B) plates at different conditions (No wash, Wash 0 min, Wash 60 min, Wash 180 min) after incubation with 500 nM probe for 1 h (n=3). *Represents the same images that are presented in Figures 2 and S1. (C) Fluorescent images (10X) of BDL-E5 and CDy1 on AiPS3 colonies on MEF- (A) and MG-coated (B) plates at different conditions (No wash, Wash 0 min, Wash 60 min) after incubation with 500 nM probe for 1 h (n=3). Scale bar represents 100 µm.

**Figure S3**

(A) (i)-(iv) Histogram (FACS) showing unstained populations of cells used as the control for FACS performed in Figure 4. (B) Fluorescence images of BDL-E5, TRA-1-60 and transmitted light (TL) images showing iPS colonies derived from ASC4 14 dpn BDL-E5^+^ (i) and BDL-E5^-^ (ii) cells at passage 0 (4X) and passage 4 (10X) (n=3). Scale bar represents 100 µm. (C) Graph showing average number of iPS colonies from BDL-E5^+^ and BDL-E5^-^ cell populations at 14 dpn in ASC4 at passage 0 (n=3). ***p < 0.001 denotes statistical significance.

**Figure S4**

(A) Representative graphs showing gene expression of *LIN28* (i), *NANOG* (ii), *Activin A* (iii) and *TGF-β1* (iv) in RNA isolated from AiPS4, ASC4, BDL-E5^+^ and BDL-E5^-^ cells of ASC4 at 14 dpn. *p < 0.05 and ***p < 0.001 denote significance compared with AiPS4; ^^p < 0.01 denotes significance compared with BDL-E5^+^ (n=3). (B) (i) Fluorescence images (10X) of TUJ1, SMA, AFP, DAPI and TL of cells following spontaneous differentiation of EBs generated from BDL-E5^+^ DPSC1. (ii)-(v) Representative graphs showing gene expression of *GATA2, SMA, AFP* and *SOX7* in RNA isolated from DiPS1 and spontaneously differentiated cells from EBs formed from BDL-E5^+^ iPS cells. ***p < 0.001 and ****p < 0.0001 denote significance compared with DiPS1 (n=3). (C) Phase contrast (PC) and fluorescent images of BDL-E5 and TRA-1-60 of reprogramming SC-ASC S16 and DPSC2 on Geltrex^TM^-coated Cytodex 3 microcarriers at 14 dpn (10X) and 21 dpn (20X). Scale bar represents 100 µm. (D) Fluorescent images of reprogramming DPSC2 on MG coated chamber slides at 7 dpn (n=3). These images are zoomed in and cropped from 20X images to clearly show the stains and their overlap; green – markers for Endoplasmic Reticulum (ER), Golgi, Lysosome, or Mitochondria; red – BDL-E5; blue – Hoechst 33342.

**Figure S5**

(A) DPSC1 was reprogrammed with the traditional method involving retroviral OCT4, SOX2, KLF4 and C-MYC, and plated onto the MEF feeder layer. Cells were co-stained with BDL-E5 (yellow), TRA-1-60 (red) and Hoechst 33342 (blue) in the indicated day post-infection (dpi). (B) BJ fibroblasts were transduced with lentiviral OCT4, SOX2, KLF4 and C-MYC in the presence or absence of A83-01 (0.3 μM) and stained at 8 dpi. The image is merged from 9 independent fields. (C) BJ fibroblasts transduced above were stained with BDL-E5 followed by cell fixation and immunostaining with TRA-1-60 at 21 dpi.

**Figure S6**

(A) Pathway analysis using Ingenuity Systems (Qiagen) shows representation of the top networks and canonical pathways between BDL-E5^+^ and BDL-E5^-^ cells. The molecular and cellular functions that were differentially expressed in BDL-E5^+^ and BDL-E5^-^ cells are also represented, along with the p values. (B) Metascape gene analysis was performed on <http://metascape.org> and the enriched clusters between BDL-E5^+^ vs. BDL-E5^-^ cells are represented here.

**Figure S7**

(A) Graph representing signal-to-noise ratios (arbitrary fluorescence units) on comparing reprogramming (RP) versus non-reprogramming (non-RP) DPSCs (DPSC1) stained with either CDy1 or BDL-E5. The fluorescence intensity was measured using ImageJ software. 100 cells per field (10X), 10 fields per well, 3 wells per probe were measured. ****p < 0001 denotes significance between RP and non-RP cells. (B) Proliferation assay of DPSC1 incubated with BDL-E5 (500 nM) for 2 to 5 days; represented as number of viable cells per cm^2^ (n=3). (C) Proliferation assay of reprogramming DPSC1, 48 h after transfection with Scr CREB1, CREB1 OE or siCREB1; represented as number of viable cells per cm^2^ (n=3).
