# Supplementary figures and images for: Novel live cell fluorescent probe for human-induced pluripotent stem cells highlights early reprogramming population
Source: Stem Cell Res Ther. 2021 Feb 5;12:113. doi: 10.1186/s13287-021-02171-6 (PMC7866770; doi:10.1186/s13287-021-02171-6)

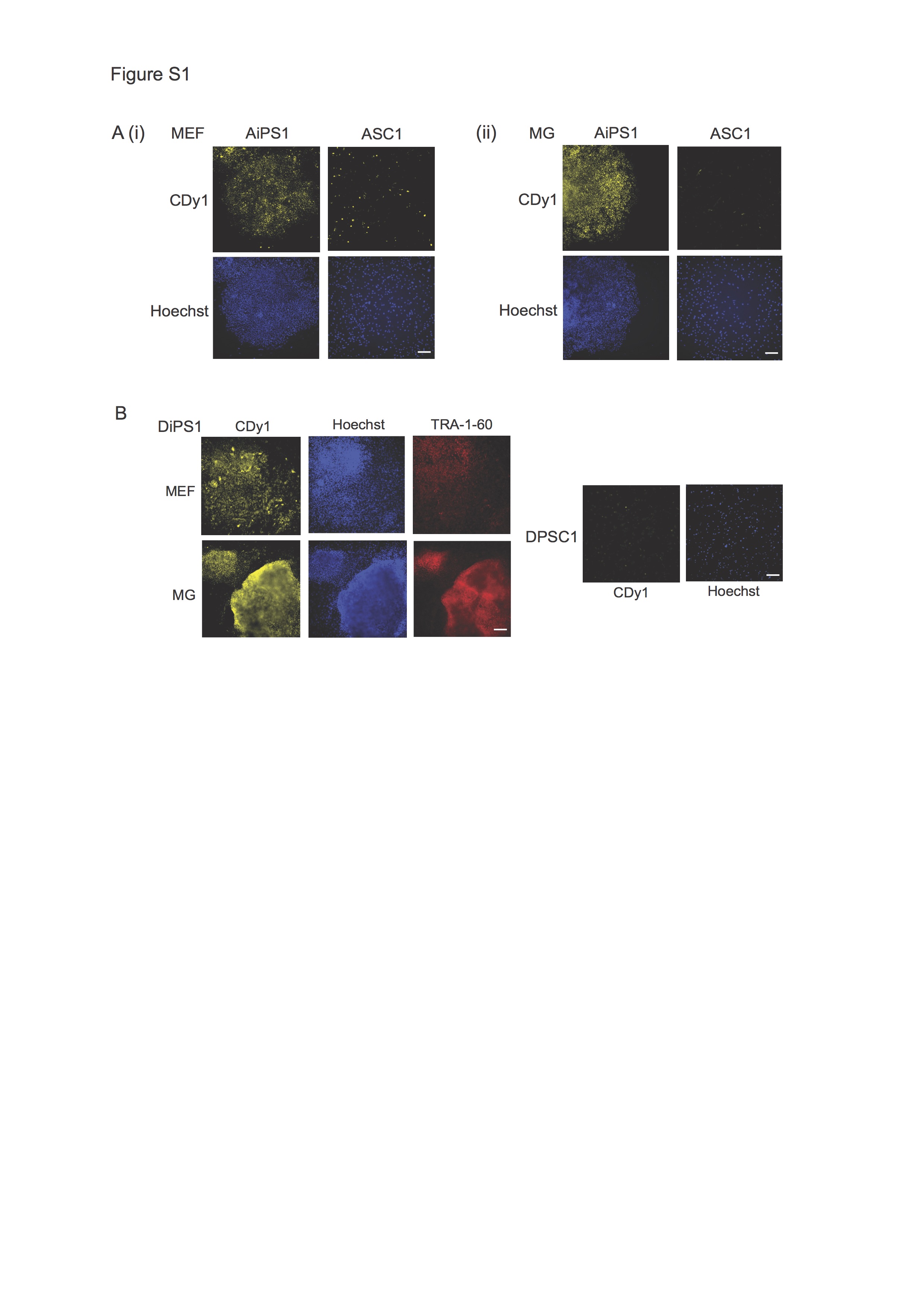

Supplement: Supplementary file 2 — Additional file 2: Figure S1. (A) Fluorescent images (10X objective) of CDy1 probe (Wash 180 min) and Hoechst on AiPS1 colonies and ASC1 on (i) MEF- and (ii) MG-coated plates from primary screening (n = 3). (B) Fluorescent images (10X) of CDy1 probe (Wash 180 min), Hoechst and TRA-1-60 on DiPS1 colonies and DPSC1 on MEF- and MG-coated plates from secondary screening. Cells were incubated with 500 nM of CDy1 in appropriate media for 1 h (n = 3). Scale bar represents 100 μm. [file 13287_2021_2171_MOESM2_ESM.jpg]

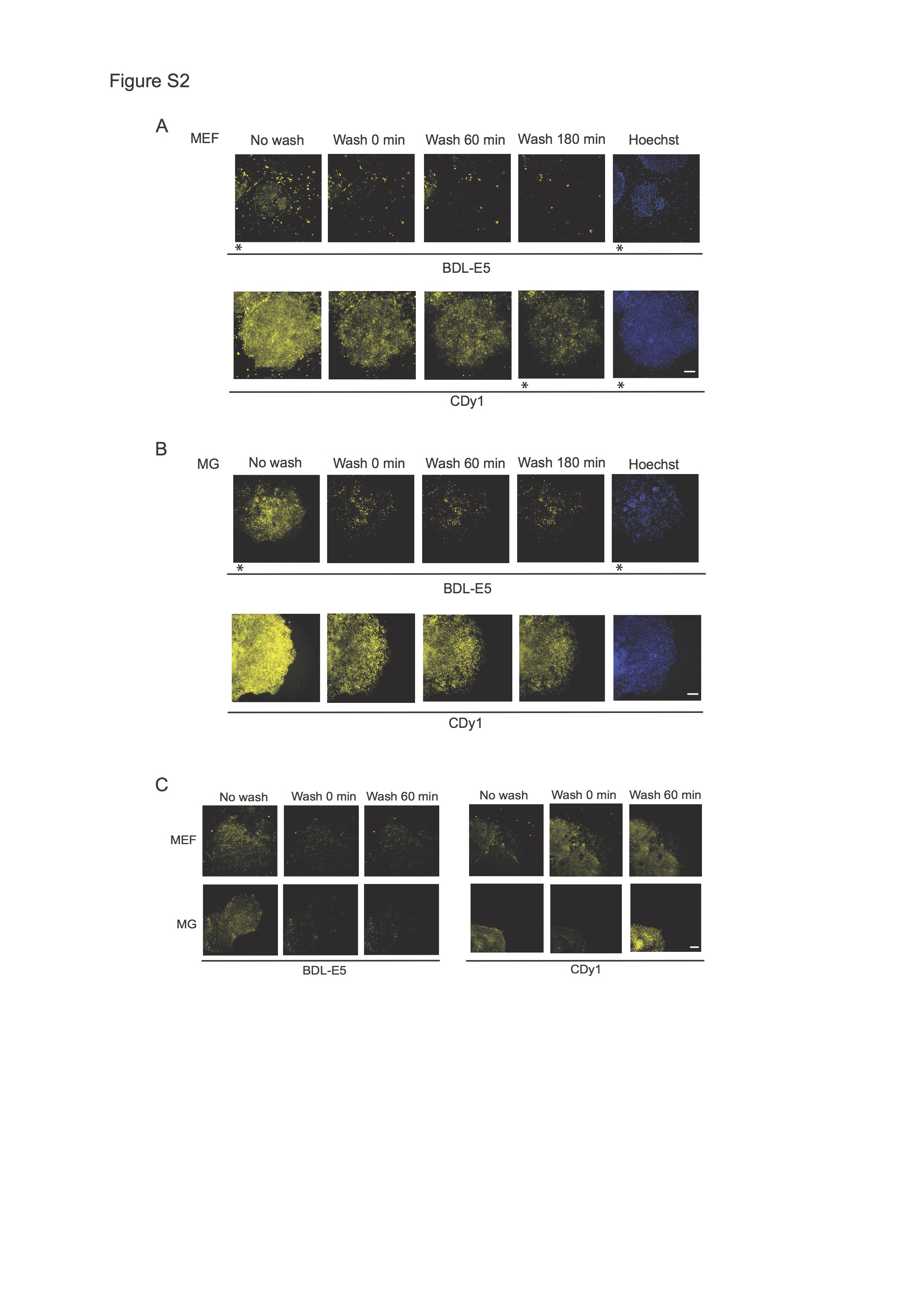

Supplement: Supplementary file 3 — Additional file 3: Figure S2. (A) Fluorescent images (10X) of BDL-E5, CDy1 and Hoechst on AiPS1 colonies on MEF- (A) and MG-coated (B) plates at different conditions (No wash, Wash 0 min, Wash 60 min, Wash 180 min) after incubation with 500 nM probe for 1 h (n = 3). *Represents the same images that are presented in Figs. 2 and S1. (C) Fluorescent images (10X) of BDL-E5 and CDy1 on AiPS3 colonies on MEF- (A) and MG-coated (B) plates at different conditions (No wash, Wash 0 min, Wash 60 min) after incubation with 500 nM probe for 1 h (n = 3). Scale bar represents 100 μm. [file 13287_2021_2171_MOESM3_ESM.jpg]

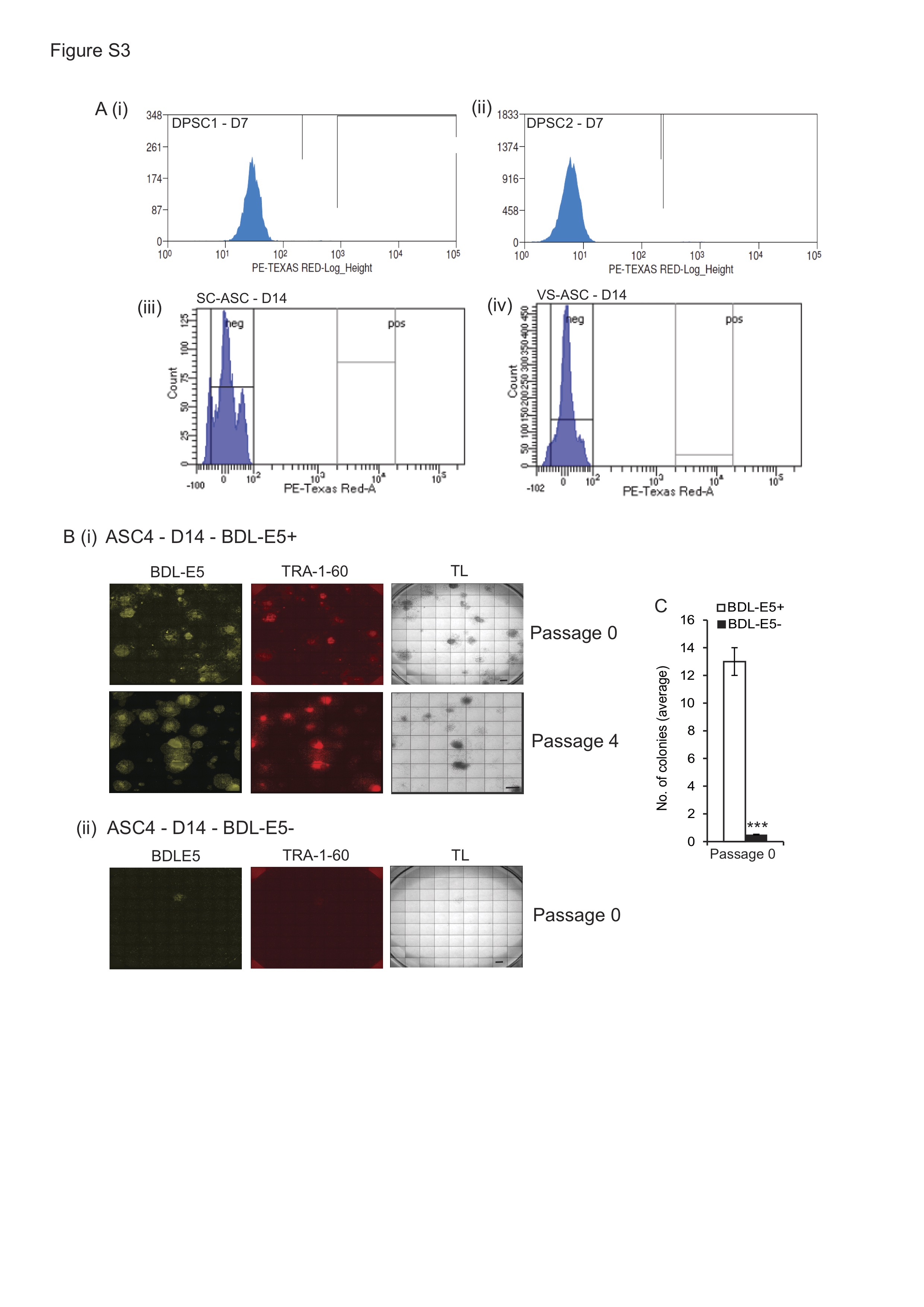

Supplement: Supplementary file 4 — Additional file 4 : Figure S3. (A) (i)-(iv) Histogram (FACS) showing unstained populations of cells used as the control for FACS performed in Fig. 4. (B) Fluorescence images of BDL-E5, TRA-1-60 and transmitted light (TL) images showing iPS colonies derived from ASC4 14 dpn BDL-E5+ (i) and BDL-E5− (ii) cells at passage 0 (4X) and passage 4 (10X) (n = 3). Scale bar represents 100 μm. (C) Graph showing average number of iPS colonies from BDL-E5+ and BDL-E5− cell populations at 14 dpn in ASC4 at passage 0 (n = 3). ***p < 0.001 denotes statistical significance. [file 13287_2021_2171_MOESM4_ESM.jpg]

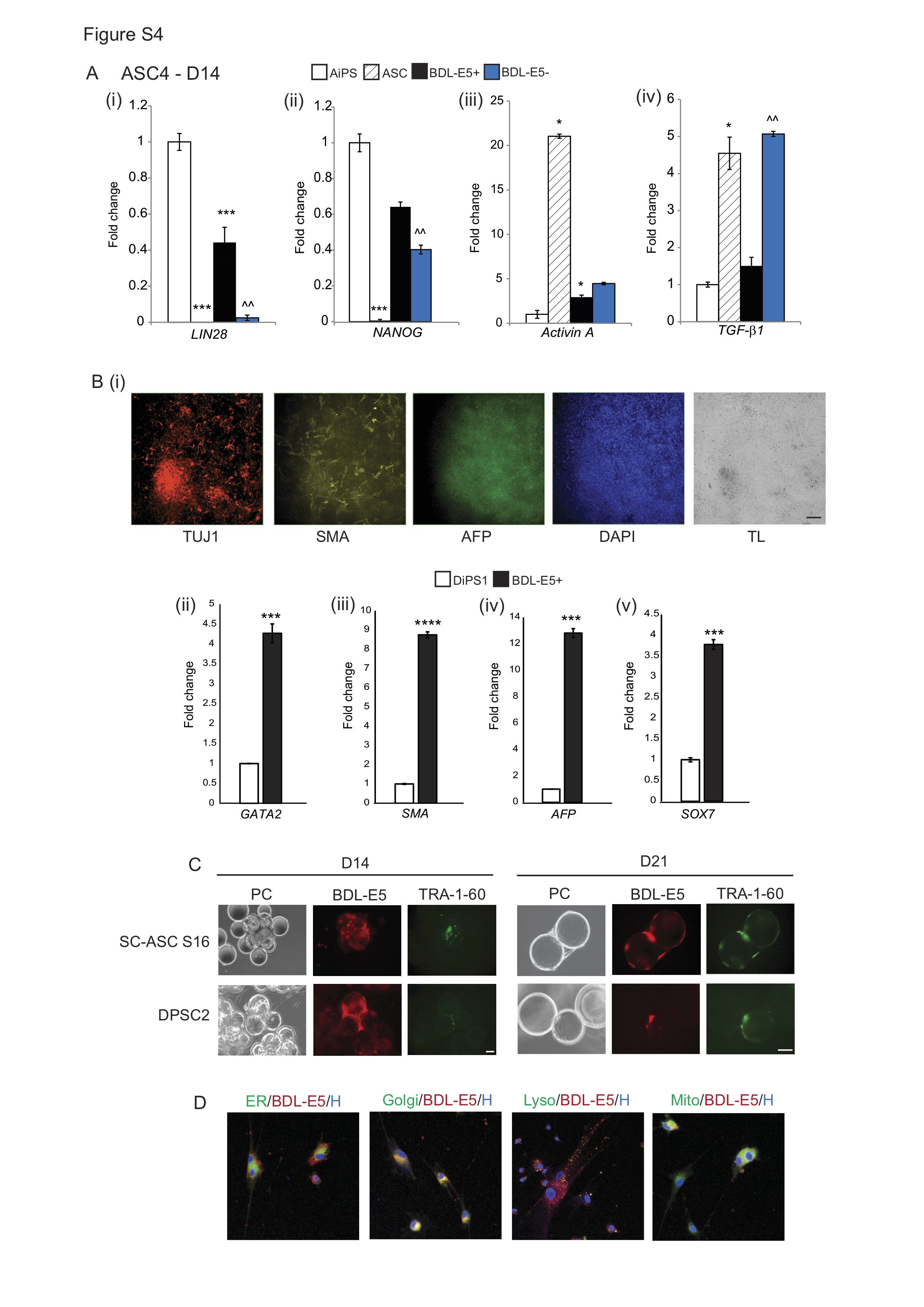

Supplement: Supplementary file 5 — Additional file 5: Figure S4. (A) Representative graphs showing gene expression of LIN28 (i), NANOG (ii), Activin A (iii) and TGF-β1 (iv) in RNA isolated from AiPS4, ASC4, BDL-E5+ and BDL-E5− cells of ASC4 at 14 dpn. *p < 0.05 and ***p < 0.001 denote significance compared with AiPS4; ^^p < 0.01 denotes significance compared with BDL-E5+ (n = 3). (B) (i) Fluorescence images (10X) of TUJ1, SMA, AFP, DAPI and TL of cells following spontaneous differentiation of EBs generated from BDL-E5+ DPSC1. (ii)-(v) Representative graphs showing gene expression of GATA2, SMA, AFP and SOX7 in RNA isolated from DiPS1 and spontaneously differentiated cells from EBs formed from BDL-E5+ iPS cells. ***p < 0.001 and ****p < 0.0001 denote significance compared with DiPS1 (n = 3). (C) Phase contrast (PC) and fluorescent images of BDL-E5 and TRA-1-60 of reprogramming SC-ASC S16 and DPSC2 on Geltrex™-coated Cytodex 3 microcarriers at 14 dpn (10X) and 21 dpn (20X). Scale bar represents 100 μm. (D) Fluorescent images of reprogramming DPSC2 on MG coated chamber slides at 7 dpn (n = 3). These images are zoomed in and cropped from 20X images to clearly show the stains and their overlap; green – markers for Endoplasmic Reticulum (ER), Golgi, Lysosome, or Mitochondria; red – BDL-E5; blue – Hoechst 33342. [file 13287_2021_2171_MOESM5_ESM.jpg]

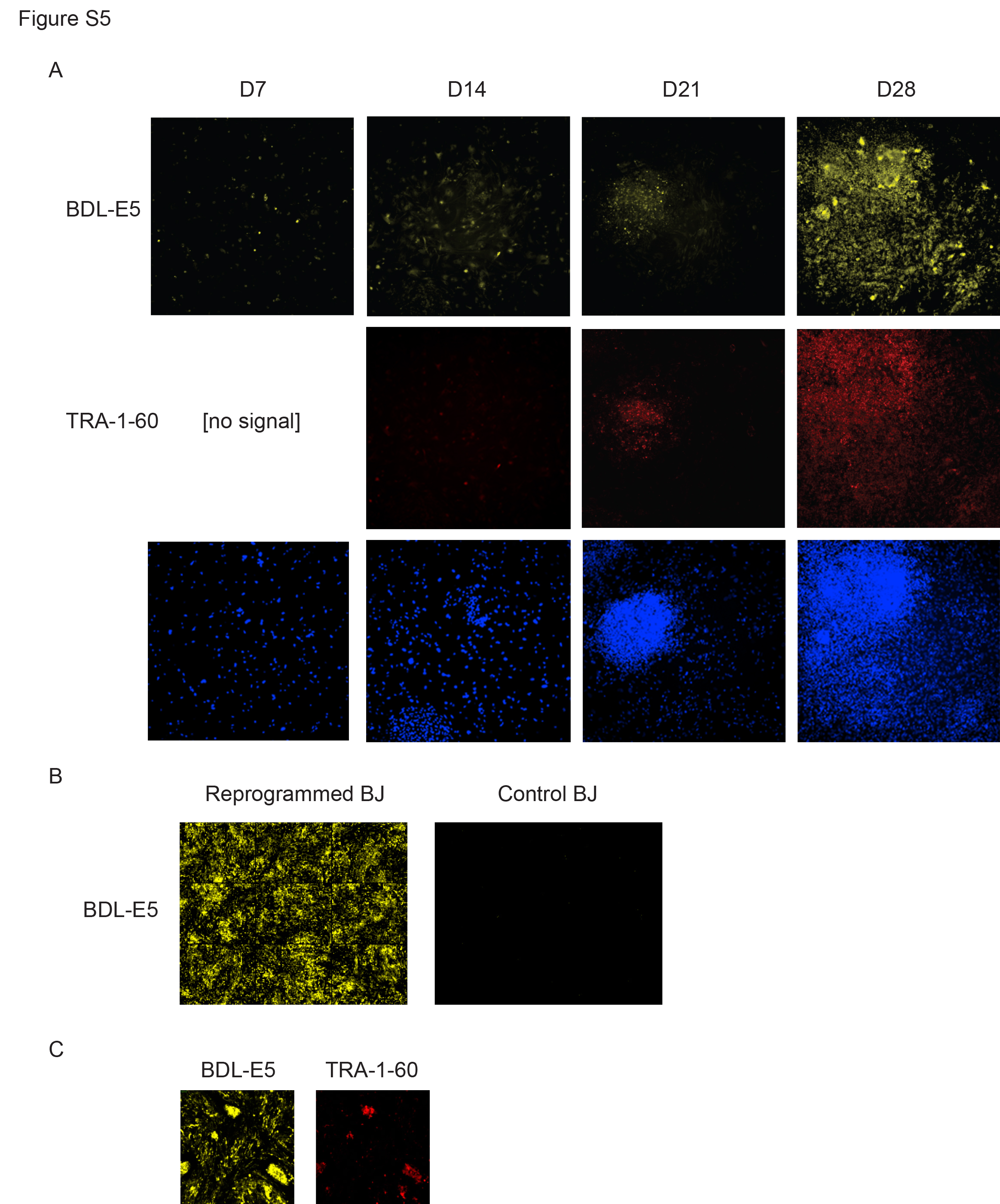

Supplement: Supplementary file 6 — Additional file 6:. Figure S5. (A) DPSC1 was reprogrammed with the traditional method involving retroviral OCT4, SOX2, KLF4 and C-MYC, and plated onto the MEF feeder layer. Cells were co-stained with BDL-E5 (yellow), TRA-1-60 (red) and Hoechst 33342 (blue) in the indicated day post-infection (dpi). (B) BJ fibroblasts were transduced with lentiviral OCT4, SOX2, KLF4 and C-MYC in the presence or absence of A83–01 (0.3 μM) and stained at 8 dpi. The image is merged from 9 independent fields. (C) BJ fibroblasts transduced above were stained with BDL-E5 followed by cell fixation and immunostaining with TRA-1-60 at 21 dpi. [file 13287_2021_2171_MOESM6_ESM.jpg]

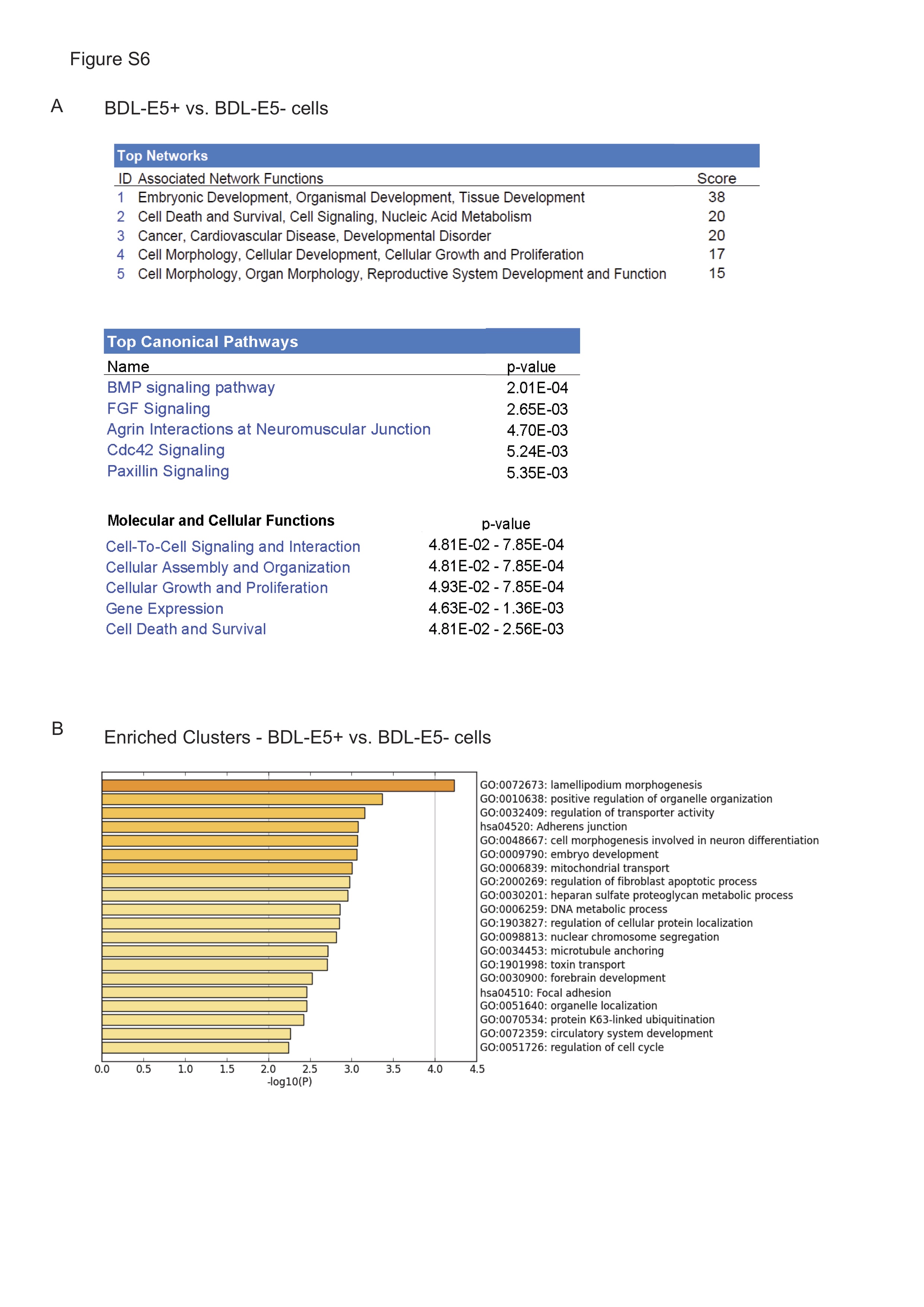

Supplement: Supplementary file 7 — Additional file 7: Figure S6. (A) Pathway analysis using Ingenuity Systems (Qiagen) shows representation of the top networks and canonical pathways between BDL-E5+ and BDL-E5− cells. The molecular and cellular functions that were differentially expressed in BDL-E5+ and BDL-E5− cells are also represented, along with the p values. (B) Metascape gene analysis was performed on http://metascape.org and the enriched clusters between BDL-E5+ vs. BDL-E5− cells are represented here. [file 13287_2021_2171_MOESM7_ESM.jpg]

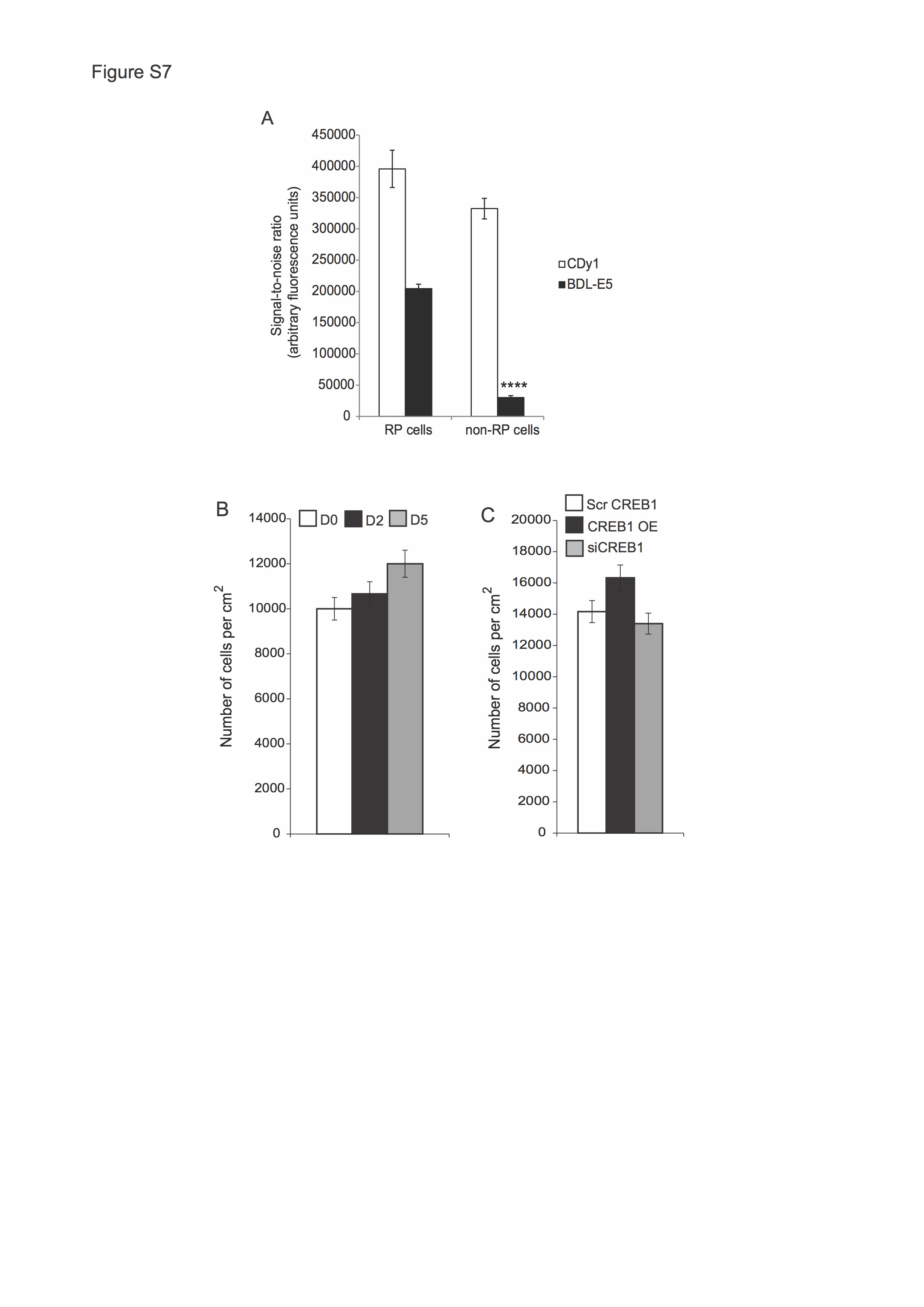

Supplement: Supplementary file 8 — Additional file 8: Figure S7. (A) Graph representing signal-to-noise ratios (arbitrary fluorescence units) on comparing reprogramming (RP) versus non-reprogramming (non-RP) DPSCs (DPSC1) stained with either CDy1 or BDL-E5. The fluorescence intensity was measured using ImageJ software. 100 cells per field (10X), 10 fields per well, 3 wells per probe were measured. ****p < 0001 denotes significance between RP and non-RP cells. (B) Proliferation assay of DPSC1 incubated with BDL-E5 (500 nM) for 2 to 5 days; represented as number of viable cells per cm2 (n = 3). (C) Proliferation assay of reprogramming DPSC1, 48 h after transfection with Scr CREB1, CREB1 OE or siCREB1; represented as number of viable cells per cm2 (n = 3). [file 13287_2021_2171_MOESM8_ESM.jpg]
