# Supplementary material for: Novel live cell fluorescent probe for human-induced pluripotent stem cells highlights early reprogramming population
Source: Stem Cell Res Ther. 2021 Feb 5;12:113. doi: 10.1186/s13287-021-02171-6 (PMC7866770; doi:10.1186/s13287-021-02171-6)
Supplement: Supplementary file 9 — Additional file 9:. Table S1. List of the 386 genes differentially expressed in DiPS, DPSC, BDL-E5+, BDL-E5-. [file 13287_2021_2171_MOESM9_ESM.pdf]

Table S1: List of the 386 genes differentially expressed in DiPS, DPSC, BDL-E5+, BDL-E5-

Gene ID	Gene Symbol	DiPS	DPSC	BDL-E5+	BDL-E5-	p value
ENSG00000029153.10	ARNTL2	0.00	0.04	0.37	4.82	0.0188
ENSG00000030304.8	MUSK	0.00	0.54	0.03	9.01	0.0089
ENSG00000059378.8	PARP12	0.00	19.17	9.55	86.06	0.0148
ENSG00000068724.11	TTC7A	0.00	15.83	1.33	40.91	0.04115
ENSG00000069869.11	NEDD4	0.00	5.41	5.13	42.48	0.00865
ENSG00000076258.5	FMO4	0.00	9.28	0.00	1.25	0.00815
ENSG00000090975.8	PITPNM2	0.00	8.28	0.19	6.26	0.02695
ENSG00000102078.11	SLC25A14	0.00	54.00	1.34	146.61	0.001
ENSG00000109667.7	SLC2A9	0.00	0.00	1.26	0.00	5.00E-05
ENSG00000117152.9	RGS4	0.00	0.00	0.45	0.00	0.0056
ENSG00000119725.13	ZNF410	0.00	0.26	30.07	0.61	0.0031
ENSG00000123243.10	ITIH5	0.00	1.29	0.00	0.64	0.00055
ENSG00000130382.7	MLLT1	0.00	16.41	0.00	104.61	5.00E-05
ENSG00000134007.3	ADAM20	0.00	0.00	5.27	0.00	0.02535
ENSG00000134539.12	KLRD1	0.00	0.00	0.54	0.00	0.0143
ENSG00000135362.9	PRR5L	0.00	5.56	0.80	21.84	0.00695
ENSG00000138772.8	ANXA3	0.00	0.00	3.12	128.24	0.0193
ENSG00000141255.8	SPATA22	0.00	0.00	0.00	0.69	0.0098
ENSG00000143127.8	ITGA10	0.00	2.80	0.58	0.00	0.01655
ENSG00000148444.11	COMMD3	0.00	63.25	35.07	273.40	0.03705
ENSG00000152128.13	TMEM163	0.00	0.00	0.00	68.59	5.00E-05
ENSG00000154678.12	PDE1C	0.00	6.75	35.24	6.84	0.0365
ENSG00000158806.9	NPM2	0.00	0.00	0.00	32.06	6.00E-04
ENSG00000159314.7	ARHGAP27	0.00	0.00	0.00	2.70	0.001
ENSG00000161681.11	SHANK1	0.00	0.00	0.00	0.55	0.00055
ENSG00000164463.8	CREBRF	0.00	7.45	19.68	0.95	0.0135
ENSG00000167136.6	ENDOG	0.00	3.97	2.83	0.00	0.03695
ENSG00000167925.11	GHDC	0.00	3.88	0.03	50.77	3.00E-04
ENSG00000168405.10	CMAHP	0.00	0.01	0.00	211.45	5.00E-05
ENSG00000172733.10	PURG	0.00	0.19	0.00	4.09	0.04485
ENSG00000173083.10	HPSE	0.00	0.00	0.43	30.85	0.048
ENSG00000178685.9	PARP10	0.00	2.15	0.97	214.51	0.03005
ENSG00000183644.9	C11orf88	0.00	2.26	0.67	0.00	0.01075
ENSG00000184588.13	PDE4B	0.00	37.02	10.35	158.90	0.0108
ENSG00000186998.11	EMID1	0.00	0.00	0.00	0.40	0.0085
ENSG00000196843.11	ARID5A	0.00	46.00	4.66	131.21	0.0251
ENSG00000197182.8	FLJ27365	0.00	3.53	0.49	23.02	0.036

ENSG00000197584.7	KCNMB2	0.00	0.00	0.00	0.59	0.02015
ENSG00000198520.6	C1orf228	0.00	0.00	0.00	79.96	0.04935
ENSG00000205746.5	RP11-1212A22.1	0.00	0.00	0.00	2.11	0.0332
ENSG00000206127.6	GOLGA8O	0.00	0.92	1.75	0.00	0.04085
ENSG00000215527.3	AP005482.1	0.00	0.00	0.00	333.91	0.0039
ENSG00000218996.1	RP1-99E18.2	0.00	0.00	0.00	4.86	0.04195
ENSG00000224080.1	UBE2FP1	0.00	0.58	0.00	1.16	0.017
ENSG00000224623.1	RP11-247I13.8	0.00	0.00	2.28	0.00	0.02075
ENSG00000225383.2	SFTA1P	0.00	0.00	0.00	11.76	0.0279
ENSG00000225920.2	RIMKLBP2	0.00	0.00	2.14	0.00	0.01615
ENSG00000227953.2	RP11-439E19.3	0.00	3.29	1.70	0.00	0.0181
ENSG00000229052.2	RP11-386I23.1	0.00	0.66	0.91	0.00	0.02955
ENSG00000229124.2	VIM-AS1	0.00	0.02	0.53	0.00	0.01325
ENSG00000229325.1	ACAP2-IT1	0.00	0.00	14.51	0.00	0.03095
ENSG00000229692.3	SOS1-IT1	0.00	0.00	0.00	10.49	0.0421
ENSG00000229808.1	RP11-456P18.2	0.00	0.00	0.91	0.00	0.0258
ENSG00000230001.1	RP11-70J12.1	0.00	2.82	12.54	0.00	0.03395
ENSG00000232116.2	RP11-187C18.2	0.00	15.03	0.00	11.70	0.04865
ENSG00000234636.1	MED14-AS1	0.00	0.00	0.00	1.40	0.0281
ENSG00000237654.1	AP003025.2	0.00	1.14	0.00	22.22	0.04965
ENSG00000237803.1	LINC00211	0.00	0.00	0.00	0.29	0.03995
ENSG00000238113.2	RP11-262H14.1	0.00	0.28	0.00	3.42	0.0337
ENSG00000240695.1	RP11-102M11.1	0.00	20.15	0.00	1.77	0.03325
ENSG00000241295.1	ZBTB20-AS2	0.00	12.76	23.50	0.00	0.0494
ENSG00000242154.1	RP4-778K6.3	0.00	0.00	12.86	0.00	0.045
ENSG00000243251.4	PGBD3	0.00	9.15	13.42	0.00	0.0263
ENSG00000243305.1	RP11-362A9.3	0.00	1.72	0.00	4.01	0.01435
ENSG00000248664.1	CTC-498J12.3	0.00	0.00	0.00	0.40	0.0166
ENSG00000249593.2	CTB-46B19.2	0.00	0.00	0.32	0.00	0.0388
ENSG00000251381.2	LINC00958	0.00	0.00	0.28	0.00	0.0079
ENSG00000255139.1	AP000442.1	0.00	0.00	0.00	178.60	0.0076
ENSG00000256025.1	CACNA1C-AS4	0.00	4.99	0.00	41.02	0.0065
ENSG00000256390.1	AC092143.1	0.00	0.00	0.41	0.00	0.01655
ENSG00000256469.1	RP11-856F16.2	0.00	1.63	0.00	6.09	0.0419
ENSG00000258978.1	HIF1AP1	0.00	0.00	0.00	124.37	0.01085
ENSG00000259948.2	RP11-326A19.5	0.00	0.00	0.68	0.00	0.02955

ENSG00000260946.1	RP11-407G23.3	0.00	27.94	0.00	74.33	0.0244
ENSG00000261355.1	RP11-698N11.4	0.00	0.17	2.90	0.00	5.00E-05
ENSG00000261777.1	RP11-529K1.2	0.00	0.00	0.70	0.00	0.0238
ENSG00000262211.1	CTD-2031P19.5	0.00	0.00	4.09	0.00	0.0189
ENSG00000267395.1	AC074212.6	0.00	7.25	2.62	0.00	0.02365
ENSG00000267515.1	RP11-861E21.3	0.00	0.00	27.81	0.00	0.0282
ENSG00000267811.1	RP11-727F15.11	0.00	1.03	6.34	0.00	0.011
ENSG00000269997.1	RP11-214K3.21	0.00	0.00	0.00	211.50	0.0488
ENSG00000272533.1	SNORA28	0.00	0.00	519.97	0.00	0.0036
ENSG00000272991.1	AF129408.17	0.00	31.84	31.93	0.00	0.0139
ENSG00000273297.1	RP11-38M8.1	0.00	0.76	7.39	0.00	0.0271
ENSG00000273384.1	RP5-1098D14.1	0.00	0.00	0.00	138.30	0.01545
ENSG00000271741.1	ZMYM6	0.00	0.03	1.77	0.00	0.04815
ENSG00000250802.2	ZBED3-AS1	0.00	0.00	1.41	0.00	0.0187
ENSG00000172716.12	SLFN11	0.00	4.19	23.14	0.40	0.0229
ENSG00000196724.8	ZNF418	0.00	0.98	0.00	0.77	0.01825
ENSG00000107738.15	C10orf54	0.00	49.53	55.13	11.04	0.0382
ENSG00000155066.11	PROM2	0.00	0.00	0.00	0.42	0.00015
ENSG00000144810.11	COL8A1	0.01	24.25	27.76	110.32	0.03055
ENSG00000163412.8	EIF4E3	0.01	1.09	1.39	0.07	0.01515
ENSG00000113296.10	THBS4	0.01	0.00	0.00	12.35	0.0023
ENSG00000123552.13	USP45	0.01	2.64	17.58	1.72	0.01925
ENSG00000137809.12	ITGA11	0.01	54.05	43.73	178.38	0.03325
ENSG00000175787.12	ZNF169	0.02	0.01	0.00	2.61	0.0192
ENSG00000105605.3	CACNG7	0.02	0.05	0.00	0.43	0.0316
ENSG00000198690.5	FAN1	0.02	0.49	2.89	0.25	0.03
ENSG00000142794.14	NBPF3	0.02	4.98	0.05	0.37	0.01015
ENSG00000006283.13	CACNA1G	0.03	0.04	0.00	0.58	0.0013
ENSG00000132256.14	TRIM5	0.03	37.19	1.75	38.68	0.0078
ENSG00000213073.4	RP11-288H12.3	0.03	0.00	0.00	36.39	0.00625
ENSG00000214176.5	PLEKHM1P	0.04	5.89	0.01	4.55	0.0222
ENSG00000135297.11	MTO1	0.04	0.16	0.87	34.48	0.0013
ENSG00000259571.1	BLID	0.04	104.78	2.91	0.00	0.03145
ENSG00000110756.13	HPS5	0.05	0.26	33.82	6.38	0.04325
ENSG00000239713.3	APOBEC3G	0.05	0.13	3.51	0.04	0.03025
ENSG00000159403.11	C1R	0.06	1182.24	205.29	848.86	0.0167
ENSG00000112769.14	LAMA4	0.07	121.77	38.18	340.15	0.00175
ENSG00000142330.15	CAPN10	0.07	30.00	0.78	165.69	5.00E-05

ENSG00000256594.3	RP11-705C15.2	0.07	0.66	0.00	4.84	0.0411
ENSG00000138639.13	ARHGAP24	0.07	0.00	85.90	0.34	0.00665
ENSG00000181938.9	GINS3	0.08	0.07	0.00	0.64	0.0057
ENSG00000219470.1	RP3-337H4.6	0.08	0.17	0.83	0.00	0.0459
ENSG00000272899.1	RP11-309L24.9	0.08	0.15	9.22	0.00	0.00605
ENSG00000134802.13	SLC43A3	0.09	0.14	1.48	0.06	0.0071
ENSG00000204991.6	SPIRE2	0.09	3.73	0.00	0.37	0.00115
ENSG00000261552.1	RP11-264B17.5	0.09	27.02	0.00	64.44	0.0378
ENSG00000235865.2	GSN-AS1	0.10	5.55	0.08	9.57	0.04845
ENSG00000183023.14	SLC8A1	0.11	19.12	3.02	65.61	0.0257
ENSG00000146373.12	RNF217	0.11	8.19	1.31	6.25	0.02805
ENSG00000121964.10	GTDC1	0.11	4.26	2.52	76.83	0.0119
ENSG00000203836.7	NBPF24	0.13	0.04	0.00	2.79	0.02085
ENSG00000175984.10	DENND2C	0.15	0.01	0.48	9.77	0.0095
ENSG00000119681.7	LTBP2	0.16	8.83	5.10	44.40	0.01055
ENSG00000269242.1	CTD-2192J16.22	0.18	7.03	35.91	0.00	0.02355
ENSG00000183655.11	KLHL25	0.18	0.14	0.00	1.15	0.0423
ENSG00000196159.7	FAT4	0.18	8.16	4.10	16.21	0.03155
ENSG00000167972.9	ABCA3	0.20	0.00	0.00	2.58	0.0303
ENSG00000140471.12	LINS	0.21	78.54	0.50	82.48	0.01345
ENSG00000235034.2	C19orf81	0.22	2.90	0.00	0.74	0.0489
ENSG00000138134.7	STAMBPL1	0.22	0.01	0.00	7.50	0.03875
ENSG00000151883.12	PARP8	0.23	0.00	0.01	0.26	0.00025
ENSG00000183405.5	RPS7P1	0.23	0.21	0.54	0.00	0.03545
ENSG00000213707.2	HMGB1P10	0.23	0.48	0.00	5.47	0.01645
ENSG00000204084.8	INPP5B	0.23	0.54	0.65	17.81	0.03345
ENSG00000160469.12	BRSK1	0.24	0.00	0.00	1.25	0.0152
ENSG00000072518.16	MARK2	0.26	0.18	1.14	24.73	0.02035
ENSG00000146263.7	MMS22L	0.27	0.25	0.00	5.45	5.00E-05
ENSG00000021645.13	NRXN3	0.30	0.02	8.17	0.00	5.00E-05
ENSG00000185278.10	ZBTB37	0.30	2.64	0.01	0.59	0.02725
ENSG00000139926.11	FRMD6	0.35	26.83	55.29	458.21	0.00925
ENSG00000223953.3	C1QTNF5	0.36	204.94	201.46	1600.38	0.00135
ENSG00000166311.5	SMPD1	0.38	94.15	112.34	585.07	0.02725
ENSG00000129657.10	SEC14L1	0.40	19.75	73.50	19.64	0.03895
ENSG00000105483.12	CARD8	0.45	22.43	0.66	14.91	0.02
ENSG00000161958.6	FGF11	0.51	1.71	0.33	18.23	0.0171
ENSG00000167615.12	LENG8	0.54	24.86	1.75	177.96	0.01025
ENSG00000160828.13	STAG3L2	0.58	9.10	0.27	5.50	0.0252
ENSG00000260793.2	RP5-882C2.2	0.63	2.04	3.70	0.00	0.01455
ENSG00000106648.9	GALNTL5	0.64	0.06	0.00	0.67	0.0041

ENSG00000157869.10	RAB28	0.64	1.06	14.07	73.16	0.0405
ENSG00000158773.10	USF1	0.69	13.14	13.84	0.00	0.022
ENSG00000229809.4	ZNF688	0.72	5.79	0.76	40.82	0.013
ENSG00000232931.1	LINC00342	0.73	6.33	8.56	64.96	0.02945
ENSG00000141576.10	RNF157	0.84	0.00	0.51	0.01	0.04825
ENSG00000255248.2	RP11-166D19.1	0.86	138.45	33.59	207.41	0.0227
ENSG00000118762.3	PKD2	0.86	28.76	39.41	8.30	0.0392
ENSG00000147457.9	CHMP7	0.88	11.38	37.02	4.23	0.0461
ENSG00000106608.12	URGCP	0.89	1.17	51.04	0.35	0.0022
ENSG00000162928.8	PEX13	0.95	4.92	4.36	146.84	0.0077
ENSG00000090674.11	MCOLN1	1.05	60.52	0.76	19.86	0.0332
ENSG00000073711.6	PPP2R3A	1.11	6.57	4.59	29.75	0.04325
ENSG00000229153.1	EPHA1-AS1	1.12	0.24	5.52	0.00	0.00925
ENSG00000232586.1	RP11-46A10.4	1.16	0.06	0.00	0.58	0.01455
ENSG00000122481.12	RWDD3	1.21	63.16	39.82	225.43	0.0445
ENSG00000136720.6	HS6ST1	1.21	60.37	23.29	123.76	0.0454
ENSG00000188130.9	MAPK12	1.29	51.40	65.20	1.23	0.0202
ENSG00000133250.9	ZNF414	1.31	0.32	0.00	24.34	0.0045
ENSG00000160352.11	ZNF714	1.32	0.24	0.00	0.97	0.0136
ENSG00000172725.9	CORO1B	1.34	1.67	19.94	1.33	0.0099
ENSG00000236526.1	RP4-742J24.2	1.35	12.53	1.22	0.00	0.0318
ENSG00000150712.6	MTMR12	1.35	5.44	6.96	0.39	0.02425
ENSG00000074527.7	NTN4	1.36	22.30	203.79	47.63	0.03745
ENSG00000197016.7	ZNF470	1.52	0.08	0.13	4.08	0.0473
ENSG00000164338.5	UTP15	1.56	2.37	1.61	57.46	0.0041
ENSG00000206560.6	ANKRD28	1.66	65.12	5.10	79.01	0.006
ENSG00000075539.9	FRYL	1.85	170.14	5.05	44.67	0.02035
ENSG00000268093.1	AC022154.7	1.88	1.09	0.00	0.50	0.0292
ENSG00000125962.10	ARMCX5	1.92	0.11	1.52	59.32	0.03555
ENSG00000129911.4	KLF16	1.93	3.55	0.99	32.26	0.04305
ENSG00000164877.14	MICALL2	1.96	62.11	32.67	229.73	0.0311
ENSG00000257647.1	RP11-701H24.3	2.00	0.94	0.00	10.99	0.02645
ENSG00000115966.12	ATF2	2.09	7.12	41.34	5.69	0.0267
ENSG00000160216.14	AGPAT3	2.12	4.83	5.54	47.29	0.02025
ENSG00000256525.2	POLG2	2.16	10.31	0.43	15.15	0.0276
ENSG00000149929.11	HIRIP3	2.26	4.78	25.38	0.03	0.0474
ENSG00000174684.6	B3GNT1	2.30	39.47	17.05	105.05	0.0474
ENSG00000105559.7	PLEKHA4	2.40	28.99	8.83	83.57	0.02915
ENSG00000187609.11	EXD3	2.49	30.13	4.28	118.89	0.00825
ENSG00000185046.14	ANKS1B	2.49	0.51	0.44	0.00	0.0165
ENSG00000184381.14	PLA2G6	2.51	0.19	0.00	1.43	0.00255
ENSG00000136436.10	CALCOCO2	2.66	22.13	46.79	333.30	0.0178
ENSG00000213918.6	DNASE1	2.66	8.69	16.15	1.03	0.04625

ENSG00000161395.8	PGAP3	2.77	4.93	0.68	54.65	0.03815
ENSG00000204196.4	AC011737.2	2.78	2.52	0.75	99.18	0.02925
ENSG00000103111.10	MON1B	2.83	22.08	44.23	1.76	0.0113
ENSG00000269279.1	AL136376.1	2.91	4.08	0.00	5.41	0.01205
ENSG00000139579.8	NABP2	2.94	6.26	0.77	47.39	0.03925
ENSG00000154370.9	TRIM11	3.17	9.59	0.73	30.51	0.0225
ENSG00000104081.9	BMF	3.20	0.45	0.03	47.46	3.00E-04
ENSG00000145012.8	LPP	3.56	10.43	10.98	120.27	0.0096
ENSG00000113716.8	HMGXB3	3.67	1.67	0.70	31.38	0.02915
ENSG00000241258.2	CRCP	3.73	38.44	21.51	2.48	0.0467
ENSG00000198380.8	GFPT1	3.79	4.53	9.62	93.28	0.0223
ENSG00000166912.12	MTMR10	3.90	8.82	5.29	33.69	0.0355
ENSG00000267542.1	RP11-697E22.1	3.95	11.63	12.97	0.00	0.0377
ENSG00000253251.2	CTC-534A2.2	4.15	5.41	0.00	3.86	0.04345
ENSG00000138035.10	PNPT1	4.37	0.72	0.87	19.99	0.0243
ENSG00000105321.8	CCDC9	4.37	0.02	1.11	0.00	0.007
ENSG00000103260.4	METRN	4.43	48.99	2.57	133.57	0.008
ENSG00000269388.1	AC018755.16	4.48	0.00	0.00	1.31	0.0316
ENSG00000198954.4	KIAA1279	4.61	19.18	8.65	70.08	0.0416
ENSG00000075420.8	FNDC3B	4.80	20.14	40.04	230.59	0.0327
ENSG00000157353.12	FUK	4.81	5.11	0.47	34.19	0.0492
ENSG00000256904.1	A2ML1-AS2	4.91	0.00	0.00	1.29	0.01695
ENSG00000177873.8	ZNF619	5.02	0.02	0.00	1.40	0.0155
ENSG00000125846.11	ZNF133	5.12	2.29	0.71	34.67	0.00535
ENSG00000118518.11	RNF146	5.16	7.44	10.78	121.79	0.01285
ENSG00000160055.15	TMEM234	5.22	0.97	6.59	0.19	0.04965
ENSG00000132004.8	FBXW9	5.31	21.06	2.55	67.37	0.03545
ENSG00000131724.6	IL13RA1	5.32	9.09	21.38	4.19	0.0234
ENSG00000196810.4	CTBP1-AS2	5.33	14.28	0.12	82.99	0.00365
ENSG00000186862.13	PDZD7	5.49	1.83	0.15	4.38	0.04035
ENSG00000243335.4	KCTD7	5.51	0.13	0.36	13.44	0.0414
ENSG00000078246.11	TULP3	5.68	4.64	5.79	194.84	0.02755
ENSG00000198055.6	GRK6	5.69	1.72	0.07	5.69	0.03345
ENSG00000149930.13	TAOK2	6.12	3.42	0.55	14.52	0.0332
ENSG00000140367.7	UBE2Q2	6.24	16.84	3.26	21.11	0.04405
ENSG00000175866.11	BAIAP2	6.27	3.32	57.96	4.18	0.00785
ENSG00000167257.6	RNF214	6.44	4.30	1.46	15.98	0.02625
ENSG00000175567.4	UCP2	6.48	0.00	0.00	1.83	0.02095
ENSG00000033178.8	UBA6	6.52	24.99	7.56	61.16	0.01225
ENSG00000157456.3	CCNB2	6.99	0.00	0.00	1.33	0.04925
ENSG00000145860.7	RNF145	7.09	17.06	7.28	92.04	0.03475
ENSG00000146243.9	IRAK1BP1	7.16	1.61	6.18	0.07	0.03085
ENSG00000130669.13	PAK4	7.20	2.21	13.55	0.93	0.02925

ENSG00000180881.15	CAPS2	7.38	0.45	0.10	18.70	0.00205
ENSG00000079739.11	PGM1	7.57	15.02	30.84	179.18	0.03445
ENSG00000108830.7	RND2	7.63	0.00	0.00	0.58	0.04305
ENSG00000168528.7	SERINC2	8.13	12.66	23.44	193.70	0.03645
ENSG00000131196.13	NFATC1	8.17	2.59	0.93	47.76	8.00E-04
ENSG00000131791.6	PRKAB2	8.24	10.26	10.18	69.19	0.0279
ENSG00000176105.9	YES1	8.75	7.29	5.68	33.89	0.0442
ENSG00000100479.8	POLE2	8.90	5.62	11.80	0.00	0.0459
ENSG00000124782.15	RREB1	8.96	25.10	1.97	89.52	0.02475
ENSG00000151746.9	BICD1	9.06	1.76	0.61	11.99	0.02745
ENSG00000110422.7	HIPK3	9.20	10.84	5.34	24.44	0.049
ENSG00000168758.6	SEMA4C	9.32	0.83	0.40	0.00	0.01885
ENSG00000107779.7	BMPR1A	9.82	2.90	1.69	13.06	0.0123
ENSG00000117713.13	ARID1A	9.88	5.75	2.74	27.83	0.0214
ENSG00000151348.9	EXT2	9.93	57.52	38.28	9.35	0.03815
ENSG00000001631.10	KRIT1	10.04	7.02	5.06	134.21	0.0125
ENSG00000177885.9	GRB2	10.04	88.12	31.28	338.39	0.03615
ENSG00000131323.10	TRAF3	10.26	3.50	3.35	0.07	0.028
ENSG00000110075.10	PPP6R3	10.72	23.86	16.20	87.31	0.024
ENSG00000163935.9	SFMBT1	11.68	2.11	0.21	89.49	0.01245
ENSG00000006468.9	ETV1	11.78	0.00	0.00	8.05	5.00E-05
ENSG00000184347.10	SLIT3	11.81	12.31	16.97	171.65	0.04765
ENSG00000119689.10	DLST	11.92	1.88	30.21	0.07	0.00345
ENSG00000181027.6	FKRP	12.13	4.73	1.48	106.76	0.04255
ENSG00000113312.6	TTC1	12.40	126.88	354.60	72.10	0.023
ENSG00000177943.9	MAMDC4	12.55	7.26	0.00	1.00	0.0257
ENSG00000105127.4	AKAP8	12.66	15.52	1.54	38.75	0.02535
ENSG00000125459.10	MSTO1	12.88	1.59	0.19	5.84	0.0181
ENSG00000161202.13	DVL3	12.95	18.30	2.19	51.52	0.01915
ENSG00000121152.5	NCAPH	14.46	0.00	0.00	63.91	0.01595
ENSG00000156970.8	BUB1B	14.58	0.00	0.06	2.81	0.047
ENSG00000138190.12	EXOC6	14.73	0.09	0.10	10.43	0.0127
ENSG00000153250.13	RBMS1	15.14	144.93	48.51	235.58	0.01575
ENSG00000124155.12	PIGT	15.15	109.11	95.81	353.88	0.04185
ENSG00000196526.6	AFAP1	15.19	26.76	5.47	92.03	0.0186
ENSG00000170310.10	STX8	16.50	40.57	267.05	1088.17	0.0152
ENSG00000145014.13	TMEM44	18.58	28.86	14.34	0.73	0.0469
ENSG00000088833.13	NSFL1C	18.81	16.25	87.23	9.00	0.042
ENSG00000127580.11	WDR24	19.13	6.16	0.35	36.69	0.04805
ENSG00000196313.7	POM121	19.33	2.06	0.11	39.20	0.0261
ENSG00000103168.12	TAF1C	19.36	21.51	0.89	26.80	0.0283
ENSG00000135506.11	OS9	19.36	68.85	29.56	242.73	0.02475
ENSG00000113734.13	BNIP1	19.53	2.07	21.33	0.67	0.04895

ENSG00000164543.5	STK17A	19.79	2.70	3.01	74.86	0.0051
ENSG00000163006.7	CCDC138	20.21	0.19	0.25	0.00	0.0038
ENSG00000151657.7	KIN	20.34	66.80	11.57	124.07	0.0316
ENSG00000126787.8	DLGAP5	20.49	0.00	0.00	0.62	0.01385
ENSG00000137942.12	FNBP1L	20.80	14.61	6.42	36.33	0.04555
ENSG00000158195.6	WASF2	20.87	99.75	79.55	679.26	0.03235
ENSG00000243725.2	TTC4	21.15	4.71	9.86	78.68	0.02515
ENSG00000168916.11	ZNF608	21.36	5.43	0.00	0.85	0.00165
ENSG00000133812.10	SBF2	21.42	45.34	39.90	6.14	0.0488
ENSG00000106012.13	IQCE	21.84	55.60	3.61	47.81	0.0439
ENSG00000139971.11	C14orf37	22.26	9.61	1.15	50.49	0.0086
ENSG00000138867.12	GUCD1	22.36	48.68	10.17	224.51	0.04415
ENSG00000267228.2	IER3IP1	22.96	32.14	7.42	0.00	0.01955
ENSG00000176390.10	CRLF3	23.22	3.67	0.06	6.89	0.00585
ENSG00000101290.9	CDS2	23.78	0.94	51.41	3.45	0.03045
ENSG00000165030.3	NFIL3	23.84	32.66	30.14	219.23	0.0146
ENSG00000198363.11	ASPH	23.84	230.74	326.46	97.56	0.019
ENSG00000062650.13	WAPAL	24.14	56.28	9.24	121.79	0.02625
ENSG00000172273.8	HINFP	24.42	16.38	0.06	0.26	0.04175
ENSG00000224019.1	RPL21P32	24.50	0.00	0.00	173.08	0.0054
ENSG00000145194.13	ECE2	24.63	33.05	0.18	16.46	0.04065
ENSG00000141985.5	SH3GL1	24.78	49.28	15.28	145.87	0.01315
ENSG00000157895.7	C12orf43	25.94	44.58	1.25	15.65	0.00735
ENSG00000138663.4	COPS4	26.31	22.05	31.53	202.21	0.0262
ENSG00000169504.10	CLIC4	26.70	140.30	205.12	48.45	0.01695
ENSG00000132676.11	DAP3	27.14	114.80	48.07	317.13	0.01545
ENSG00000103275.14	UBE2I	28.48	34.42	17.49	91.88	0.0274
ENSG00000122482.16	ZNF644	28.54	18.23	27.17	3.57	0.04385
ENSG00000127946.12	HIP1	29.77	5.83	3.13	75.14	0.0379
ENSG00000118260.10	CREB1	31.96	4.70	42.52	3.49	0.0027
ENSG00000152413.10	HOMER1	32.53	4.81	1.83	90.78	0.00105
ENSG00000090686.11	USP48	33.75	118.84	39.13	133.69	0.0307
ENSG00000075131.5	TIPIN	33.93	1.21	0.17	3.45	0.0392
ENSG00000124574.10	ABCC10	34.82	0.80	5.09	0.00	0.02555
ENSG00000136153.15	LMO7	35.40	384.16	247.03	1215.23	0.0112
ENSG00000034677.7	RNF19A	37.93	22.98	13.14	163.02	0.03895
ENSG00000063244.8	U2AF2	38.77	3.04	15.08	1.20	0.043
ENSG00000152942.14	RAD17	39.05	6.99	5.55	158.97	0.0465
ENSG00000113658.12	SMAD5	39.06	60.62	18.06	251.89	0.04935
ENSG00000066923.13	STAG3	41.21	1.55	0.00	2.21	0.0047
ENSG00000010318.15	PHF7	44.31	5.43	0.00	3.88	0.012
ENSG00000157500.6	APPL1	44.31	62.47	47.78	4.28	0.01785
ENSG00000144524.13	COPS7B	45.83	34.73	7.04	73.72	0.00975
ENSG00000116679.11	IVNS1ABP	46.20	33.51	14.92	125.37	0.01845
ENSG00000134363.7	FST	46.86	248.92	116.59	481.74	0.0361

ENSG00000168724.10	DNAJC21	47.19	158.17	33.90	356.35	0.0107
ENSG00000081320.6	STK17B	47.87	21.00	65.30	6.08	0.02835
ENSG00000077782.15	FGFR1	53.88	115.95	47.08	226.61	0.019
ENSG00000131037.10	EPS8L1	54.02	0.00	0.00	68.85	5.00E-05
ENSG00000100796.13	SMEK1	54.56	29.85	9.80	113.19	0.007
ENSG00000112576.8	CCND3	56.84	12.85	41.94	216.48	0.04555
ENSG00000077312.4	SNRPA	57.78	5.97	3.16	100.41	0.0107
ENSG00000038382.13	TRIO	58.79	62.20	10.88	116.43	0.0124
ENSG00000117616.13	C1orf63	59.63	357.82	133.16	732.99	0.0166
ENSG00000112531.12	QKI	61.47	6.84	5.88	47.98	0.0346
ENSG00000185864.12	NPIPB4	61.65	24.39	3.66	36.08	0.0051
ENSG00000140632.12	GLYR1	62.41	25.16	3.82	127.73	9.00E-04
ENSG00000064607.12	SUGP2	66.35	40.94	18.89	2.14	0.03335
ENSG00000163697.12	APBB2	68.17	9.71	44.92	5.15	0.0026
ENSG00000100836.6	PABPN1	68.71	136.49	36.31	186.46	0.0399
ENSG00000214021.11	TTLL3	70.98	33.52	2.43	439.85	0.0016
ENSG00000103091.10	WDR59	76.24	8.34	2.46	55.56	0.04075
ENSG00000242114.1	MTFP1	76.61	0.00	1.52	147.89	0.0217
ENSG00000116809.7	ZBTB17	79.34	10.40	1.01	30.96	0.0256
ENSG00000054654.11	SYNE2	81.62	2.70	0.50	3.68	0.0446
ENSG00000145016.9	KIAA0226	89.15	11.86	9.78	139.03	0.0266
ENSG00000109920.8	FNBP4	92.79	42.91	33.64	230.39	0.0158
ENSG00000119048.3	UBE2B	103.42	39.37	100.96	682.64	0.0443
ENSG00000172889.11	EGFL7	106.66	0.63	1.09	230.19	0.01655
ENSG00000101115.8	SALL4	106.90	5.55	0.00	74.06	5.00E-05
ENSG00000089006.12	SNX5	107.25	102.70	44.24	405.60	0.01565
ENSG00000223745.3	RP4-717I23.3	108.07	11.50	55.98	5.28	0.029
ENSG00000078808.12	SDF4	111.39	113.08	19.25	419.99	0.0034
ENSG00000150753.7	CCT5	114.70	49.14	79.81	294.12	0.0456
ENSG00000074054.13	CLASP1	116.42	4.10	21.39	548.20	0.0067
ENSG00000176022.3	B3GALT6	121.05	36.08	29.74	238.61	0.0345
ENSG00000118058.16	KMT2A	131.38	28.29	9.16	86.35	0.0133
ENSG00000157110.11	RBPMS	135.68	51.42	17.47	188.47	0.01675
ENSG00000114857.13	NKTR	137.91	92.83	23.91	169.09	0.0072
ENSG00000197122.7	SRC	140.76	2.22	0.25	8.21	0.02595
ENSG00000163714.13	U2SURP	151.90	162.07	42.15	384.12	0.0478
ENSG00000184465.11	WDR27	159.07	5.88	0.34	23.27	4.00E-04
ENSG00000118420.12	UBE3D	164.38	2.03	3.43	0.00	0.01815
ENSG00000078674.13	PCM1	168.30	78.81	20.62	277.38	0.0087
ENSG00000227540.1	RP11-152N13.5	192.77	0.00	0.00	3.80	0.03325
ENSG00000088448.10	ANKRD10	203.31	220.78	22.86	349.50	0.00545

ENSG00000167508.6	MVD	206.88	29.59	1.56	103.48	0.0225
ENSG00000113013.8	HSPA9	212.44	86.01	70.16	304.00	0.02625
ENSG00000111215.7	PRR4	216.81	153.65	62.96	0.91	0.0136
ENSG00000101104.8	PABPC1L	217.65	0.20	6.83	140.08	0.04645
ENSG00000108395.9	TRIM37	221.03	46.46	9.74	142.01	0.00515
ENSG00000129932.3	DOHH	231.87	3.48	0.74	0.00	0.0136
ENSG00000091136.9	LAMB1	235.00	123.61	46.18	317.48	0.0161
ENSG00000168000.10	BSCL2	262.44	172.02	41.28	475.11	0.0411
ENSG00000103855.13	CD276	263.15	70.14	43.84	307.87	0.0259
ENSG00000076242.10	MLH1	351.00	1.09	12.59	179.35	0.00955
ENSG00000164292.8	RHOBTB3	360.56	48.32	128.06	23.71	0.0216
ENSG00000132589.11	FLOT2	506.43	122.30	66.52	257.29	0.0479
ENSG00000117523.11	PRRC2C	584.21	187.20	62.17	380.76	0.04055
ENSG00000111642.10	CHD4	826.30	694.96	78.30	1169.69	0.0155
ENSG00000100401.15	RANGAP1	896.77	106.57	36.05	257.91	0.03255
ENSG00000141002.14	TCF25	1709.60	676.19	229.02	1118.74	0.03775
ENSG00000198804.2	MT-CO1	2186.47	10593.20	1150.07	6626.71	0.0111
ENSG00000101361.10	NOP56	2251.98	158.79	31.64	220.42	0.04485
ENSG00000214548.10	MEG3	3738.76	600.30	96.15	384.80	0.02685
