# Supplementary material for: Novel live cell fluorescent probe for human-induced pluripotent stem cells highlights early reprogramming population
Source: Stem Cell Res Ther. 2021 Feb 5;12:113. doi: 10.1186/s13287-021-02171-6 (PMC7866770; doi:10.1186/s13287-021-02171-6)
Supplement: Supplementary file 10 — Additional file 10:. Table S2. Real time qPCR primers. [file 13287_2021_2171_MOESM10_ESM.pdf]

Table S2: Real time qPCR primers

Gene	Forward 5' to 3'	Reverse 5' to 3'
GAPDH	CAAGGTCATCCATGACAACTTTG	GGCCATCCACAGTCTTCTGG
Activin A	CTCGGAGATCATCACGTTTG	CCTTGGAAATCTCGAAGTGC
LIN28	GAAGCGCAGATCAAAAGGAG	GCTGATGCTCTGGCAGAAGT
Nanog	CCAACATCCTGAACCTCAGC	GCTATTCTTCGGCCAGTTG
DPPA2	TGGTGTCAACAACTCGGTTTG	CTCGAACATCGCTGTAATCTGG
TGF-β1	GCAGCACGTGGAGCTGTA	CAGCCGGTTGCTGAGGTA
FN1	CTGGCCGAAAATACATTGTAAA	CCACAGTCGGGTCAGGAG
DNMT3B	TACACAGACGTGTCCAACATGGGC	GGATGCCTTCAGGAATCACACCTC
GDF3	AAATGTTTGTGTTGCGGTCA	TCTGGCACAGGTGTCTTCAG
Cdh1	GAAGGTGACAGAGCCTCTGGAT	GATCGGTTACCGTGATCAAAATC
EpCAM1	TGTGTGCGTGGGA	TTCAAGATTGGTAAAGCCAGT
ZEB1	AGCAGTGAAAGAGAAGGGAATGC	GGTCCTCTTCAGGTGCCTCAG
ZEB2	CGCAGCACATGAATCACAGG	CGTATCGTTTCGGGATCCGT
Snail1	TCTGAGGCCAAGGATCTCCA	CATTCGGGAGAAGGTCCGAG
Snail2	TCATCTTTGGGGCGAGTGAG	TCCTTGAAGCAACCAGGGTC
CREB1	ATTGGAAGGAAAGGGGAGGG	GGCTTGAACACATCTTGGCA
PRKAB2	CAGTAGAGTGGGGCAGGAAA	TCCCATTTCACATCTGGGCT
GATA2	AAGGCTCGTTCCTGTTCAGA	GGCATTGCACAGGTAGTGG
SOX7	GAGCAGTGTGGACACGTACC	GTCCAGGGGAGACATTTCAG
SMA	CTGTTCCAGCCATCCTTCAT	TCATGATGCTGTTGTAGGTGGT
AFP	AAGAATTTCAGCATGATTTTCCA	CACCCACTTCATGGTTGCTA
